# Spermine oxidase induces DNA damage and sensitizes fusion negative rhabdomyosarcoma cells to irradiation

**DOI:** 10.3389/fcell.2023.1061570

**Published:** 2023-01-23

**Authors:** Clara Perrone, Silvia Pomella, Matteo Cassandri, Michele Pezzella, Stefano Giuliani, Tecla Gasperi, Antonella Porrazzo, Anna Alisi, Anna Pastore, Silvia Codenotti, Alessandro Fanzani, Giovanni Barillari, Libenzio Adrian Conti, Biagio De Angelis, Concetta Quintarelli, Paolo Mariottini, Franco Locatelli, Francesco Marampon, Rossella Rota, Manuela Cervelli

**Affiliations:** ^1^ Department of Hematology and Oncology, Cell and Gene Therapy, Bambino Gesù Children’s Hospital, IRCCS, Rome, Italy; ^2^ Department of Science, “Department of Excellence 2018-2022”, University of Rome “Roma Tre”, Rome, Italy; ^3^ Department of Clinical Sciences and Translational Medicine, University of Rome “Tor Vergata”, Rome, Italy; ^4^ Department of Radiological Sciences, Oncology and Anatomical Pathology, Sapienza University of Rome, Rome, Italy; ^5^ Biostructures and Biosystems National Institute (INBB), Rome, Italy; ^6^ Unit of Molecular Genetics of Complex Phenotypes, Bambino Gesù Children’s Hospital, IRCCS, Rome, Italy; ^7^ Research Unit of Diagnostical and Management Innovations, Bambino Gesù Children’s Hospital, IRCCS, Rome, Italy; ^8^ Department of Molecular and Translational Medicine, University of Brescia, Brescia, Italy; ^9^ Confocal Microscopy Core Facility, Bambino Gesù Children’s Hospital, IRCCS, Rome, Italy; ^10^ Department of Clinical Medicine and Surgery, Federico II University of Naples, Naples, Italy; ^11^ Department of Life Sciences and Public Health, Catholic University of the Sacred Heart, Rome, Italy

**Keywords:** rhabdomyosarcoma, polyamine pathway, SmOx, DNA damage, radiotherapy, radioresistance, combination therapy, soft tissue sarcoma

## Abstract

Rhabdomyosarcoma (RMS) is a pediatric myogenic soft tissue sarcoma that includes fusion-positive (FP) and fusion-negative (FN) molecular subtypes. FP-RMS expresses PAX3-FOXO1 fusion protein and often shows dismal prognosis. FN-RMS shows cytogenetic abnormalities and frequently harbors RAS pathway mutations. Despite the multimodal heavy chemo and radiation therapeutic regimens, high risk metastatic/recurrent FN-RMS shows a 5-year survival less than 30% due to poor sensitivity to chemo-radiotherapy. Therefore, the identification of novel targets is needed. Polyamines (PAs) such as putrescine (PUT), spermidine (SPD) and spermine (SPM) are low-molecular-mass highly charged molecules whose intracellular levels are strictly modulated by specific enzymes. Among the latter, spermine oxidase (SMOX) regulates polyamine catabolism oxidizing SPM to SPD, which impacts cellular processes such as apoptosis and DNA damage response. Here we report that low SMOX levels are associated with a worse outcome in FN-RMS, but not in FP-RMS, patients. Consistently, SMOX expression is downregulated in FN-RMS cell lines as compared to normal myoblasts. Moreover, SMOX transcript levels are reduced FN-RMS cells differentiation, being indirectly downregulated by the muscle transcription factor MYOD. Noteworthy, forced expression of SMOX in two cell lines derived from high-risk FN-RMS: 1) reduces SPM and upregulates SPD levels; 2) induces G0/G1 cell cycle arrest followed by apoptosis; 3) impairs anchorage-independent and tumor spheroids growth; 4) inhibits cell migration; 5) increases γH2AX levels and foci formation indicative of DNA damage. In addition, forced expression of SMOX and irradiation synergize at activating ATM and DNA-PKCs, and at inducing γH2AX expression and foci formation, which suggests an enhancement in DNA damage response. Irradiated SMOX-overexpressing FN-RMS cells also show significant decrease in both colony formation capacity and spheroids growth with respect to single approaches. Thus, our results unveil a role for SMOX as inhibitor of tumorigenicity of FN-RMS cells *in vitro*. In conclusion, our *in vitro* results suggest that SMOX induction could be a potential combinatorial approach to sensitize FN-RMS to ionizing radiation and deserve further in-depth studies.

## Introduction

Rhabdomyosarcoma (RMS) is a soft tissue sarcoma of childhood that represents 8%–9% of all pediatric solid tumors. RMS cells are sought to derive from mesenchymal precursors that express Myogenic Regulatory transcription Factors (MRFs) such as MYOD and Myogenin (MYOG) but are blocked in an undifferentiated proliferative stage (Skapek et al., 2019). About 25% of RMS are characterized by chromosomal translocations: among them the most relevant one is t (2; 13) leading to the expression of the oncogenic chimeric transcription factor PAX3- FOXO1 (P3F) (Shern et al., 2014). Tumors expressing P3F are at ultra-high risk of recurrence, often being metastatic at diagnosis. FN-RMS has no distinct molecular characteristics, but most of the mutations found affect the RAS pathway genes (e.g., NRAS, KRAS, HRAS) in 5%–35% of cases (Shern et al., 2014). Moreover, TP53 mutations leading to inactivation of the p53 pathway have been detected in about 13% of FN-RMS patients and correlate with lower survival (Shern et al., 2021). Additional mutations were found in fibroblast growth factor receptor 4 (FGFR4) and phosphoinositide-3-kinase (PI3K) genes inducing the activation of the respective pathways (Shern et al., 2014; Shern et al., 2021). Despite a multi-therapeutic approach high risk FN-RMS often show a dismal prognosis with a 5-year survival less than 30% due to chemo and radiation therapies unresponsiveness (Bergeron et al., 2021). Therefore, there is an urgent need to develop novel combinatorial therapeutic approaches aimed at restoring the response to treatments. To this end, deeper knowledge of processes and pathways specifically involved in RMS biology is a fundamental step.

Polyamines (PAs), such as putrescine (PUT), spermidine (SPD) and spermine (SPM), are organic polycationic alkylamines found in all living cells. They are synthesized from L-arginine (*via* L-ornithine) and L-methionine by a series of interdependent enzymatic reactions and they are present in the cells at millimolar concentration (Wallace et al., 2003; Pegg, 2016). A schematic representation of PAs metabolism is depicted in Supplementary Figure S1. Importantly, these low-molecular-mass and highly charged molecules have bene found to be involved in several important processes such as cell growth and survival, including maintenance of protein and nucleic acid (DNA and RNA) synthesis, stabilization of chromatin structure, differentiation, apoptosis, protection from oxidative damage and regulation of different ion channels functions (Cervelli et al., 2009; Kahana, 2016; Arruabarrena-Aristorena et al., 2018; Murray Stewart et al., 2018; Igarashi and Kashiwagi, 2019).

In order to preserve the normal cell functions, PAs intracellular content must be maintained within a certain level *via* a tight control of both biosynthesis/catabolic pathways and import/export systems (Cervelli et al., 2014a; Casero et al., 2018; Murray Stewart et al., 2018).

Deregulation of PAs metabolism has been observed in several types of cancer, including medulloblastoma (D’Amico et al., 2015), carcinoma of the breast (Silvestrini et al., 1989; Manni et al., 1995), colon (Basu Roy et al., 2008; Kucharzewska et al., 2009; Snezhkina et al., 2016; Wang et al., 2017), prostate (Li et al., 2020), and cervix (Chen et al., 2018).

Spermine oxidase (SMOX) is the enzyme that catalyzes a key reaction in PA metabolism directly oxidating SPM to SPD with concomitant production of H_2_O_2_ and 3-aminopropanal (Cervelli et al., 2013). SMOX has been involved in several cellular processes such as cell drug response, apoptosis, DNA damage response (DDR) (Amendola et al., 2005; Cervelli et al., 2010; Cervelli et al., 2014a; Cervelli et al., 2014b). Further, SMOX ectopic expression in neuroblastoma cells increases oxidative DNA damage thus inducing apoptosis, and these effects tare enhanced by the exposure to ionizing radiation (Amendola et al., 2005; Bianchi et al., 2007; Amendola et al., 2013; Amendola et al., 2014).

Herein, we have investigated the role of SMOX in RMS. Our results indicate that SMOX low expression correlates with poor prognosis in FN-RMS patients. We also show that SMOX is indirectly repressed by MYOD and is downregulated in patient-derived RAS- and p53-mutated high-risk FN-RMS cell lines compared to normal myoblasts. Forced SMOX expression impairs the tumorigenic features of FN-RMS cells and promotes DNA damage, thereby sensitizing FN-RMS cells to ionizing radiation.

## Materials and methods

### Bioinformatic analyses

Association between SMOX expression and overall RMS patients’ survival was obtained using Williamson dataset (E-TABM-1202). Data has been downloaded at https://www.ebi.ac.uk/arrayexpress/experiments/E-TABM-1202/and extracted with RStudio. Kaplan-Meier curve has been obtained using R2 platform (https://hgserver1.amc.nl/cgi-bin/r2/main.cgi). The *p*-values are given by logrank test, where data is dichotomized into lowly expressed and highly expressed groups by percentiles. The best separation, smallest *p*-value is then reported, accompanied by a Kaplan Meier picture. CRISPR data for a panel of tumor cell lines were downloaded from DepMap (https://depmap.org/portal/), from the database CRISPR (Avana) Public 20Q2 (1,032 cell lines, 10 RMS cell lines, established by the Broad Institute and the Wellcome Sanger Institute) and plotted with GraphPad Prism 8. Perturbation effects have been reported as CERES score. RNA-seq data of HSMM and Trametinib-treated RD cells are from GSE52529 and GSE85170 respectively.

### Cell lines

RD (FN-RMS) cell line was obtained from American Type Culture Collection (ATCC) (Rockville, MD, United States). JR1 cell line was from Janet Shipley lab. and was authenticated by STR analysis (9 loci). RD and JR1 cells were cultured in DMEM high-glucose (Invitrogen, Carlsbad, CA, United States) supplemented with 10% fetal bovine serum (FBS), 1% of an L-glutamine solution and 1% of a penicillin-streptomycin solution. Normal Human Skeletal Muscle Myoblasts (HSMM, #CC-2580) and growth media with supplements and serum (CC-3245) were purchased from Lonza (Walkersville, MD, United States). C3H/10T1/2 murine fibroblasts (Clone 8 catalog CCL-226) were purchased from ATCC and cultured in Eagle’s Basal Medium (#2101-046, Thermofisher Scientific, Rockford, United States) supplemented with 10% FBS and 2 mM L-glutamine. All the cell lines were cultured at 37°C in a humidified atmosphere of 5% CO2/95% air. All the cell lines were regularly checked for *mycoplasma* contamination. HSMMs were cultured in according to manufacturer’s instructions.

### Real time RT-quantitative

Total RNA was extracted using TRIzol (Invitrogen, Carlsbad, CA, United States) according to the manufacturer’s protocol and as reported in (Camero et al., 2021). qRT-PCR analyses were carried out by SYBR-Green (human SMOX: For 5′-ACG​GAG​ATG​CTG​CGT​CAG​TTC​A-3′, Rev 5′-CCT​GCG​TGT​ATG​AAT​AGG​AGC​C-3′; human ß-Actin: For 5′-CAT​GGG​TCA​GAA​GGA​TTC​CTA​T-3′, Rev 5′-ATG​TCG​TCC​CAG​TTG​GT-3′; murine Smox: For 5′-ACT​CCA​AGA​ATG​GCG​TGG​C-3′, Rev 5′-CGA​CGC​TGT​TCT​GAC​TCT​C-3′; murine Gapdh: Fwd 5′-GGT​TGT​CTC​CTG​CGA​CTT​C-3′, Rev 5′-GGT​GGT​CCA​GGG​TTT​CTT​AC-3′) and TaqMan Gene Assay (Applied Biosystems, Life Technologies, Carlsbad, CA, United States: human MYOD1 (Hs02330075_g1), human GAPDH (Hs99999905_m1), murine MyoD1 (Mm00440387_m1), murine Hprt (Mm1545399_m1). The QuantStudio 3 Real-Time PCR System (Applied Biosystems) was used for the measurements. The expression fold change was calculated by the 2^−ΔΔCT^ method for each of the reference genes.

### Protein extraction and western blot

The whole-cell lysates were obtained by homogenizing cells in RIPA lysis buffer as previously described (Menna et al., 2022). Detection was performed by Pierce™ ECL Western Blotting Substrate (Thermo Scientific™) or Western Lightning ECL Pro (PerkinElmer, Waltham, MA, United States). Antibody against SMOX (SAB1101510) was obtained from Merck-Millipore Corporation, (Darmstadt, Germany). Phospho-ATM (Ser 1981) (sc-47739) and ATM (sc-377293) was from Santa Cruz Biotechnology Inc., (Santa Cruz, CA, United States); Vinculin (#V9131) was from Sigma (St Louis, MO, United States). Antibody against pospho-DNA-PKcs (Thr2609) (10B1) were obtained from Abcam (Toronto, ON, Canada). Antibodies against Phospho-Histone H2A.X (Ser139) (#9718), Histone H2AX (#2595) and all secondary antibodies were obtained from Cell Signaling (Beverly, MA, United States), α-TUBULIN (NB100-92249) was from Novus Biologicals (Littleton, CO, United States). All antibodies were used in accordance with the manufacturer’s instructions.

### Retrovirus production and cell infection

To obtain a pBABE retro vector expressing the endogenous human SMOX, the coding sequence of SMOX (iGenBankTM accession number AY033889) was amplified, and cloned into a pBABE-puro vector (Addgene #1764). Amplification was obtained using the following primers: -SMOX-1F 5′-TTT​ATA​CTC​GAG​CCT​AGA​AGG​TGA​GCA​CGG​AC-3′ and - SMOX-2R 5′-AAA​TAT​CTC​GAG​GGA​ACA​CAT​TTG​GCA​GTG​AGG-3′ and allowed to introduce XhoI restriction sites at both ends of the fragment. Amplified PCR product was restricted by XhoI (New England BioLabs, Herts, United Kingdom) and ligated with the restricted SalI (New England BioLabs, Herts, United Kingdom) pBABE-puro vector, resulting in pBABE SMOX vector (hereafter pSMOX). The accuracy of the nucleotide sequence of the recombinant pSMOX vector was verified by sequencing and then utilized to transduce FN-RMS cell lines. The pBABE-puro empty vector (hereafter pBABE) was the control vector. A pBABE retro vector expressing murine MyoD (pMyoD) (Plasmid #20917, www.addgene.org) and pBABE vector as control was used to transduce the C3H/10T1/2 murine fibroblasts. To produce the retroviral particles, 293 GP cells (kindly provided by Gian Maria Fimia, National Institute for Infectious Diseases, I.R.C.C.S. Lazzaro Spallanzani, Rome, Italy) were cultured in DMEM supplemented with 10% FBS, 1% L-glutamine and 1% penicillin-streptomycin and transiently transfected using Calcium Phosphate. After 24 h of incubation at 37°C, transfection medium was replaced with 10 mL of complete medium containing 10% FBS. Supernatant containing viral particles, collected after 72 h, was filtered through a 0.45-mm filter and was used to infect RD and JR1 cells O/N in the presence of polybrene (5 μg/mL). Cells were harvested 24, 48, and 72 h (h) after infection for subsequent experiments.

### Polyamines content determination

Polyamines content was determined as described in (Ceci et al., 2017) with minor modifications. Perchloric acid suspension (5%), supplemented with 1.7-diaminoeptane 100 μM as an internal standard, was added to cellular pellet of FN-RMS cell line transduced with pSMOX or pBABE for 20 days. Samples were sonicated in ice with Sonics Vibra-Cells to disintegrate the tissue and centrifuged at 16,100 g for 10 min. Once the supernatant was mixed with saturated Na_2_CO_3_ and acetone Dansyl chloride solution (7.5 mg/mL), was incubated overnight at room temperature protected from the light. The next day, the samples were centrifuged at 16,100 g for 15 min at 4°C and proline solution (5%) was added to the supernatant to remove the unreacted Dansyl chloride. After 30 min, PAs were extracted with toluene (100%) with vigorous vortexing and then rested for 5 min at room temperature in the dark. The organic phase was dried in a 3 Speedvac Concentrator (Savant Instrument, Inc., New York, United States) and dried Dansyl derivatives were stored at −20°C or dissolved in methanol and immediately assayed. To detect PAs content, High-performance liquid chromatography (HPLC) was performed using the Agilent 1,050 system (Agilent Technologies, Germany), with an Agilent 1,050 photodiode type detector. Continuous on-line quantification of chromatographic peaks was carried out by a fluorimeter Agilent 1,200 Spectra-Physics Model SP 4290 and a computing program software “Agilent ChemStation.” The separation of Dansyl derivatives was performed on C18 Hypersil BDS 250 × 4.6 mm at constant room temperature 22°C ± 1. Two mobile phases were used: (A) water: acetonitrile: methanol (50%:30%: 20%) and (B) acetonitrile: methanol (60%:40%) with the following elution program: 0–5 min: 72% A- 28% B; 5–47 min: 72% A- 28% B; 47–50 min: 36%A- 64% B; 50–55 min: 20% A- 80% B; 55–56 min: 15% A- 85% B; 56–75 min: 72% A- 28% B at flow rate of 1 mL/min.

### Cell cycle, apoptosis assays and migration assay

After 24 h of retroviral infection, RD and JR1 cells were fixed in cold 50% PBS/5% FBS and 50% acetone/methanol (1:4 v/v) for 1 h. Cell pellets were stained for cell cycle analysis in the dark with a solution of 0.1 mg/mL propidium iodide (ThermoFisher Scientific, Rockford, United States) and 2 mg/mL RNase (Sigma Chemical Co., St Louis, MO, United States) for 30 min at room temperature. The cells were then analyzed by flow cytometer as reported in (Cassandri et al., 2021) using a FACSCantoII equipped with a FACSDiva 6.1 CellQuestTM software (Becton Dickinson Instrument, San Josè, CA, United States). For apoptosis assay, 48 h post infection the cells were incubated with PE-conjugated Annexin V and 7-Aminoactinomycin D (7-AAD) in binding buffer for 15 min in the dark, using Annexin V apoptosis detection kit (BD Pharmingen, San Diego, CA, United States), according to manufacturer’s recommendations and reported in (Wang et al., 2021). The apoptotic cells were examined using the aforementioned flow cytometer. For migration assay, the cells were seeded at 100% of confluence on 96-well plate using Oris Cell Migration Assay Kit (#CMA1.101, Platypus Technologies, Madison, WI, United States) according to manufacturer instructions. Analysis of cell migration into the detection zone was performed after fixing and staining with Diff-Quick^®^ (460.053, Medion Diagnostic AG, Düdingen, Switzerland) as reported in (Perrone et al., 2022). The images were taken using the Leica microscope Leica DMi8 with LAS X Navigator image acquisition software.

### Soft agar colony formation assays

A total of 10^4^ RD and JR1 cells, transduced with pBABE and pSMOX, were suspended in DMEM (10% FBS) containing 0.5% agar (50,081, NuSieve GTG Agarose, Lonza, Walkersville, MD, United States. Cells were seeded on a layer of 1% agar in DMEM (10% FBS) in 6 multi-well plates as in (Pomella et al., 2021). Medium was refreshed every 2 days. On day 14, colonies were counted by microscopic inspection and images were acquired with Leica microscope Leica DMi8. Duplicate assays were carried out in three independent experiments.

### Immunofluorescence

RD and JR1 cells were fixed with 4% paraformaldehyde (PFA)/PBS for 15 min at room temperature (RT), permeabilized in 0.2% Triton X-100/PBS for 5 min at room temperature (RT), and incubated with rabbit Phospho-Histone H2A.X (Ser139) (#9718) (Cell Signaling, Beverly, MA, United States) in 1% BSA/PBS. Alexa-488 goat α-rabbit (Invitrogen) was used as secondary antibody. Cells were counterstained with DAPI and imaged using the Olympus microscope FV3000 with Olympus FV315S-SW image acquisition software.

### *In vitro* irradiation and colony formation assay

Radiation was delivered at RT using a Radgil2, an X-Ray irradiator. For DNA damage and spheroids growth analyses, the cells were transduced for 24 h with either pBABE or pSMOX retrovirus, and then irradiated with a single dose of 4Gy. For clonogenic survival assays, cells were irradiated with a single dose of 2Gy. The colony formation assay was performed as in (Rossetti et al., 2021). Three hours post irradiation, cells were counted and plated in growth medium in triplicate in 6 multi-well. Medium was refreshed every 2 days, and after 14 days, cells were fixed and stained with Diff-Quik^®^ (460.053, Medion Diagnostic AG, Düdingen, Switzerland) as manufacturer’s instruction. Colonies containing >50 cells were counted.

### Gene silencing

RD cells were transiently transfected with either human MYOD siRNA (siMYOD) (sequence: CUU​GCC​ACA​ACG​GAC​GAC​UU) or with a control scramble siRNA (siSCR) (SIC001) (Sigma-Aldrich, St Louis, MO, United States) with a final concentration of 100 nM using Oligofectamine (Invitrogen, Carlsbad, CA), according to the manufacturer’s instructions. Twenty-4 hours later, the medium was replaced with fresh growth medium supplemented with 10% FBS, 1% L-glutamine and 1% penicillin-streptomycin, and the transfected cells were harvested at different time points.

### Spheroids generation and image acquisition

For 3D tumor spheroids, RD and JR1 cells, transduced with pBABE and pSMOX retrovirus, were seeded in 100 µL of complete growth media on 96 Ultra-Low Attachment (#7007) (CORNING, New York, United States) well plates as previously described (Perrone et al., 2022). Diameters of spheroids were evaluated every 24 h using Celigo image cytometer (Nexcelom Bioscience, Lawrence, MA, United States). Six-days after seeding, diameter was determined and 3D tumor spheroids were stained using Propidium Iodide (0.1 mg/mL final concentration), Calcein AM (1 mM final concentration) and Hoechst (1:10,000). Images acquisition and analysis were performed using Celigo image cytometer (Nexcelom Bioscience, Lawrence, MA, United States).

### Statistical analysis

The data were presented as the means ± SD. Comparisons were made between the means from three independent experiments performed in three technical replicates. Student’s two tailed *t*-test and 2-way ANOVA were performed. Statistical significance was set at a two-tailed *p*-value less than 0.05. All analyses were performed with SPSS 11.5.1 for Windows Package (SPSS, Inc., 1989_2002 and LEADTOOLS 1991_2000, LEAD Technologies, Inc, Chicago, IL, United States).

## Results

### SMOX low expression in FN-RMS patients correlates with poor outcome

**FIGURE 1:**
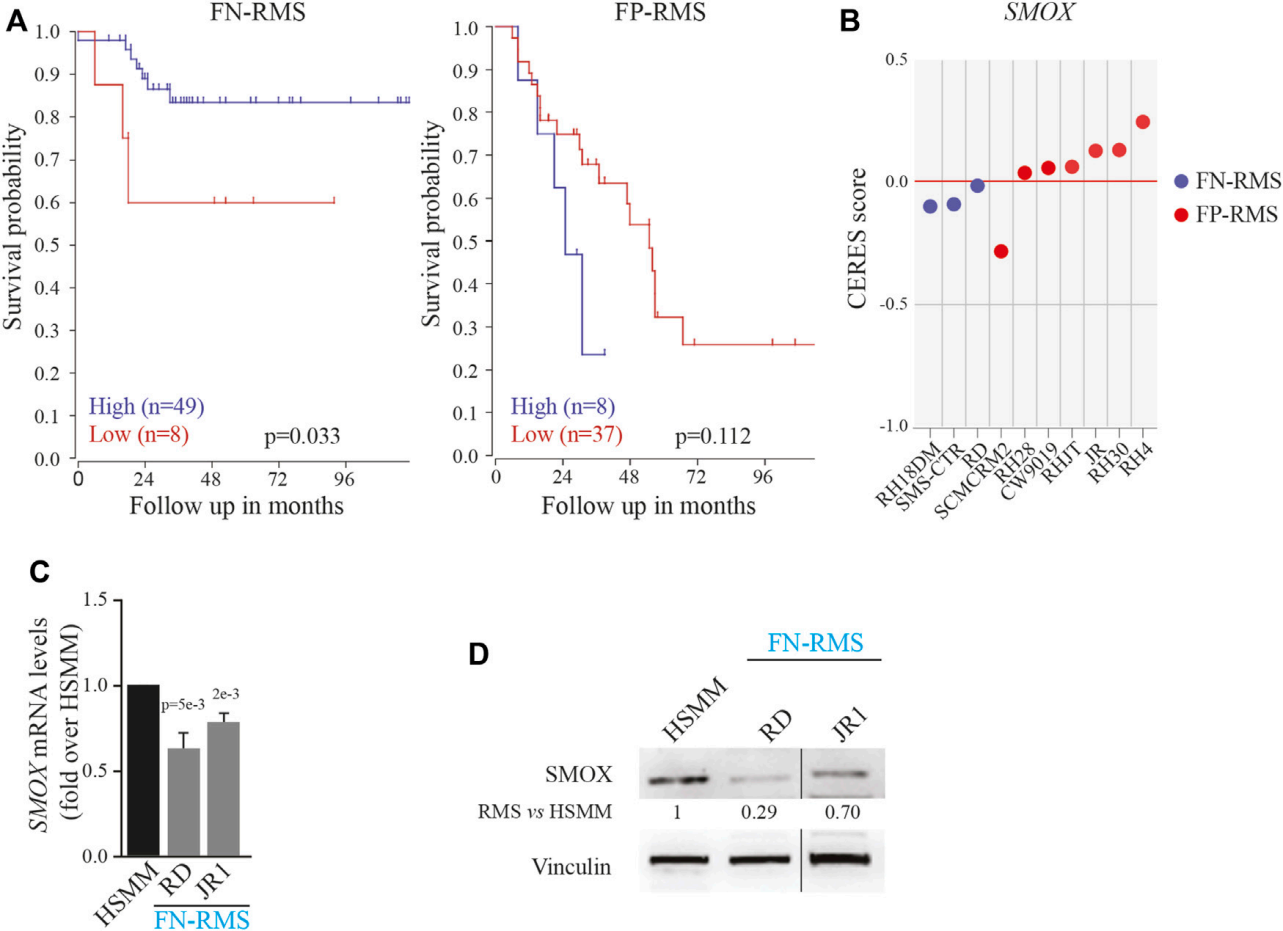
Impact of SMOX expression on RMS patients and cell lines. **(A)** Correlation between SMOX expression level [Williamson dataset (E-TABM-1202)] and FN- (*n* = 57) and FP-RMS (*n* = 45) patients’ survival. **(B)** Effects of SMOX depletion on RMS cancer cell lines obtained by DepMap (www.depmap.org). **(C)** SMOX mRNA levels (qRT- PCR) in RD and JR1 FN-RMS cell lines and in normal Human Skeletal Muscle Myoblasts (HSMM) were normalized to GAPDH levels and expressed as fold increase over HSMM values. Graph represents the mean of three independent experiments ±SD, Student two-tailed *t*-Test. Exact *p*-values are reported in the figure. **(D)** Representative western blot depicting SMOX protein levels in whole-cell lysates from RD and JR1 FN-RMS cell lines and HSMM. Vinculin levels have been used as loading control. HSMM cells have been used as control. Vertical line indicates a cropped lane.

To evaluate the impact of SMOX expression in a translational context, we analyzed the survival of FN-RMS and FP-RMS patients. Unexpectedly, as shown in Figure 1A, low expression of SMOX was associated with bad prognosis in FN-RMS patients, while the correlation was not significant in FP-RMS ones. Then, we examined the effects of SMOX depletion in RMS cell lines reported in DepMap, a publicly available dataset of 1,032 cell lines in which the gene has been knocked out by CRISPR/Cas9 (www.depmap.org). From this analysis, SMOX did not appear to be an essential gene for survival in all the investigated RMS cell lines (Figure 1B). Due to the significant link with prognosis, we assessed SMOX expression levels in the RD and JR1 cell lines, which are derived from high-risk FN-RMS, that is from recurrent tumors and harboring TP53 mutations (Shern et al., 2021), RD and JR1. SMOX transcripts and protein levels were downregulated in both cell lines compared to normal Human Skeletal Muscle Myoblasts (HSMM) as control (Figures 1C, D).

Interestingly, SMOX expression was further downregulated in RD cells treated with the MEK1/2 inhibitor Trametinib as a model of robust myogenic-like differentiation (Supplementary Figure S2A) (Yohe et al., 2018). Recently putative binding sites for the master muscle transcription factor MYOD on SMOX regulatory regions have been suggested in RD cells (Tenente et al., 2017). To gain insights into the transcriptional regulation of SMOX in our context, we forcedly expressed an exogenous murine MyoD in murine fibroblasts and noticed that SMOX levels decreased starting from 24 h post-MyoD induction (Supplementary Figure S2B). In agreement, MYOD silencing in RD cells, using validated siRNA (Gryder et al., 2017), led to SMOX upregulation both at mRNA and protein levels (Supplementary Figure S2C). When we analyzed the correlation of the two genes in a cohort of FN-RMS patients by employing a public dataset (Davicioni et al., 2006), we found a significant negative correlation between the expression of SMOX and MYOD1 (Supplementary Figure S2D).

Taken together, these data indicate that SMOX low levels could have an impact on the prognosis of FN-RMS patients and that the transcriptional downregulation of the enzyme is linked to MYOD expression in FN-RMS cell lines.

### SMOX overexpression decreases FN-RMS cell proliferation

**FIGURE 2:**
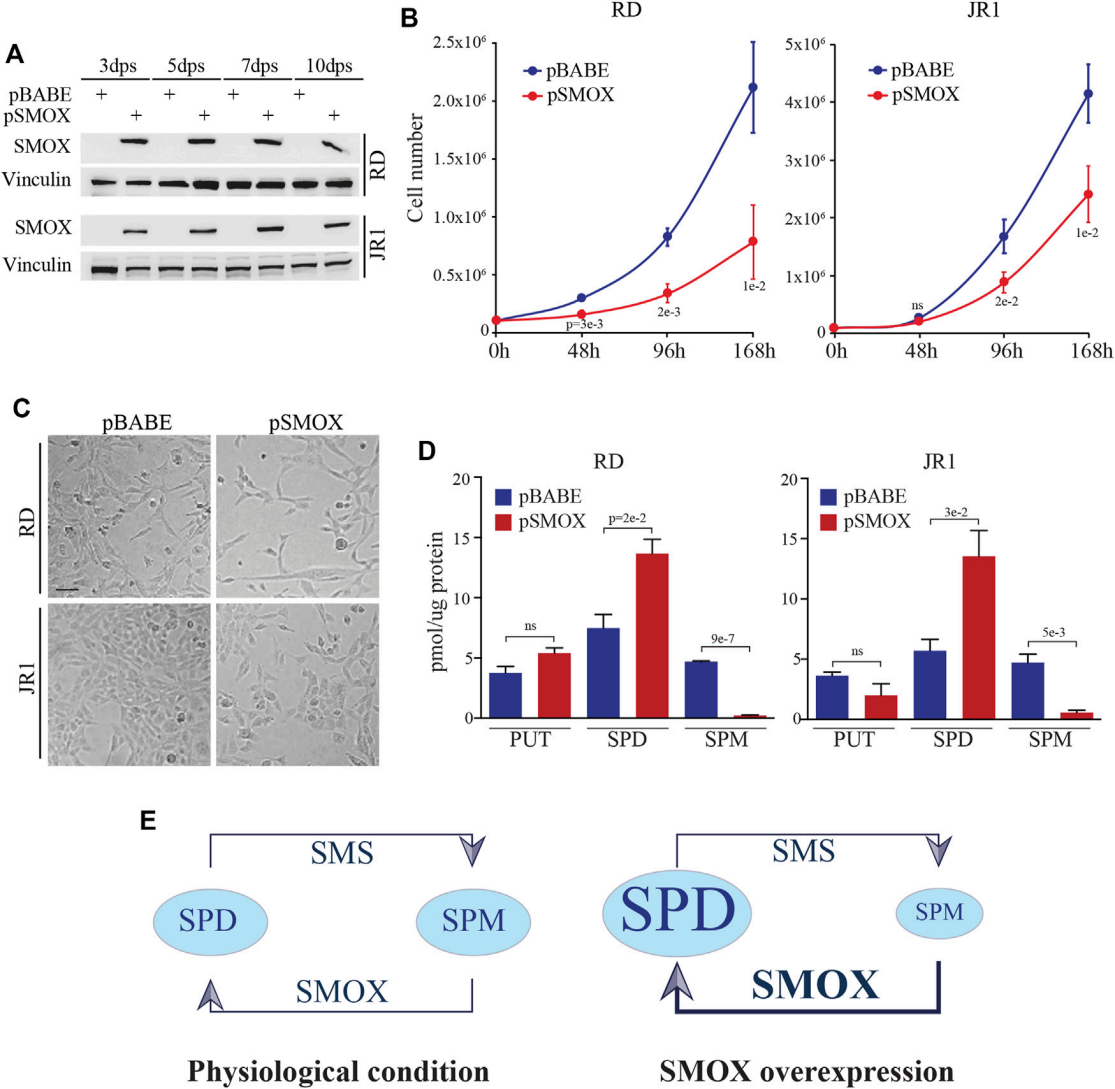
SMOX overexpression in FN-RMS cell lines reduces cell proliferation. **(A)** Representative western blot (*n* = 3 independent biological replicates) depicts SMOX overexpression at 3, 5, 7, and 10 days post selection (dps) with puromycin in RD and JR1. Vinculin was used as loading control. **(B)** Growth-curve analysis of RD and JR1 at 0, 48, 96, and 168 h post seeding. Graph represents the mean of three independent experiments ±SD, Student two-tailed *t*-Test. Exact *p*-values are reported in the figure. **(C)** Representative images of SMOX overexpressing-RD and -JR1 96 h post seeding. Scale Bar = 100 μm. **(D)** Histograms of putrescine (PUT), spermidine (SPD) and spermine (SPM) concentrations expressed as pmoles/µg of protein in SMOX overexpressing-RD and -JR1 cells. Graph represents the mean of three independent experiments ±SD, Student two-tailed *t*-Test. Exact *p*-values are reported in the figure. **(E)** Schematic representation of SPD and SPM concentration balance in our experimental condition. In physiological conditions (left) SMS and SMOX enzymes maintain the balance between SPM and SPD concentration. SMOX overexpression (right), induces an imbalance shifts making the SPD the predominant polyamine.

Given SMOX downregulation in FN-RMS patients with bad prognosis and in FN-RMS cell lines, we investigated the effects of SMOX forced expression in FN-RMS cells. To this end, RD and JR1 cells were infected with a retroviral SMOX expressing vector (pBABE SMOX, hereafter pSMOX) or with an empty vector (pBABE) as control, and cell proliferation was assessed in a time course experiment. Both cell lines overexpressing SMOX showed a significant decrease of cell growth at 96 h post infection compared to pBABE cells (Figures 2A–C). Specifically, the reduction reached 59 ± 11% in RD and 47 ± 5% in JR1 at 96 h post-seeding, while at 168 h the reduction was 64 ± 8% in RD and 42 ± 7% in JR1.

Forced expression of SMOX was functional as it resulted in an increased production of SPD early post-SMOX induction, and in a reduction of SPM levels in pSMOX compared to pBABE cells (Figures 2D, E).

Altogether, these results suggest that SMOX enzyme inhibits cell proliferation in FN-RMS cells.

### SMOX overexpression induces G0/G1 cell cycle arrest and apoptosis in FN-RMS cells

**FIGURE 3:**
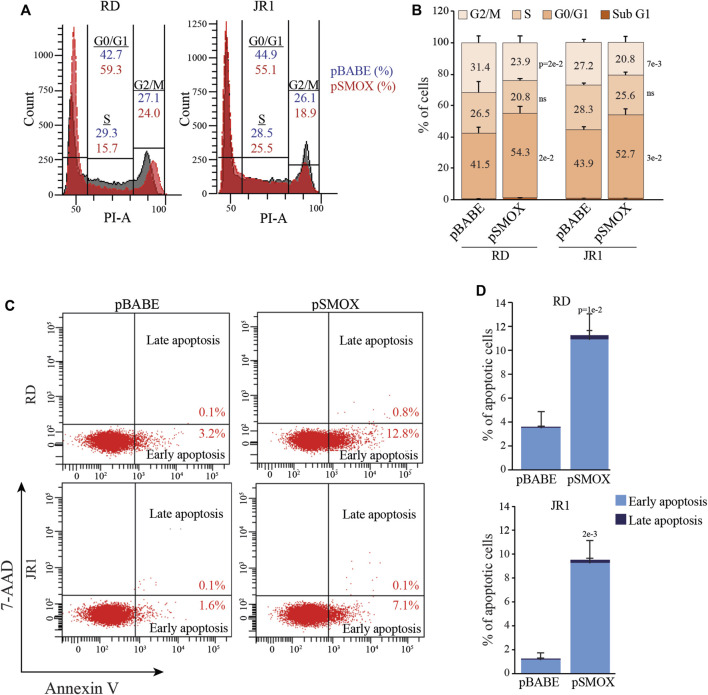
SMOX overexpression in FN-RMS cell lines induces G0/G1 cell cycle arrest and apoptosis. **(A)** Representative diagrams of flow cytometry analysis of Propidium Iodide (PI) stained RD and JR1 transduced with pBABE and pSMOX and analyzed 24 h post transduction. **(B)** Histogram depicting the percentage of SMOX overexpressing-RD and -JR1 cells in subG1, G0/G1, S, and G2/M phases. Graph represents the mean of three independent experiments ±SD, Student two-tailed *t*-Test. Exact *p*-values are reported in the figure. **(C)** Representative cytofluorimetric plots of Annexin V/7-AAD (*n* = 3 independent biological replicates) of RD and JR1 transduced with either pBABE or pSMOX and analyzed 48 h post transduction. **(D)** Histogram depicting the percentage of Annexin-V positive/7-AAD single- and double-positive RD and JR1 cells. Graphs represent the mean of three independent experiments ±SD, Student two-tailed *t*-Test. Exact *p*-values are reported in the figure.

To identify the cell cycle phase involved in decreased proliferation, we evaluated cell cycle distribution in RD and JR1 cells 24 h after transduction with pSMOX and the control vector pBABE. As reported in Figures 3A, B, SMOX overexpression determined a G0/G1 arrest in both cell lines with an increase of the percentage of cells in G0/G1 phase of 12.8 ± 5.2% in RD and 8.8 ± 2.1% in JR1 cells and a decrease of the ones in S and G2/M phases of 5.7 ± 7.1% and 7.5 ± 2.2% in RD and 2.7 ± 0.9% and 6.4 ± 1.4% in JR1, compared to pBABE.

Consistent with the fact that cell cycle arrest can induce programmed cell death, we found that the percentage of cells positive for Annexin V, a marker of apoptosis, significantly increased 48 h post-SMOX-overexpression (7.7 ± 3.6% and 8.2 ± 2.5% in RD and JR1, respectively, compared to pBABE cells) (Figures 3C, D). These data indicate that SMOX overexpression is able to arrest FN-RMS cells in G0/G1 phase with a consequent induction of apoptosis.

### SMOX overexpression reduces *in vitro* tumorigenic features in FN-RMS cells

**FIGURE 4:**
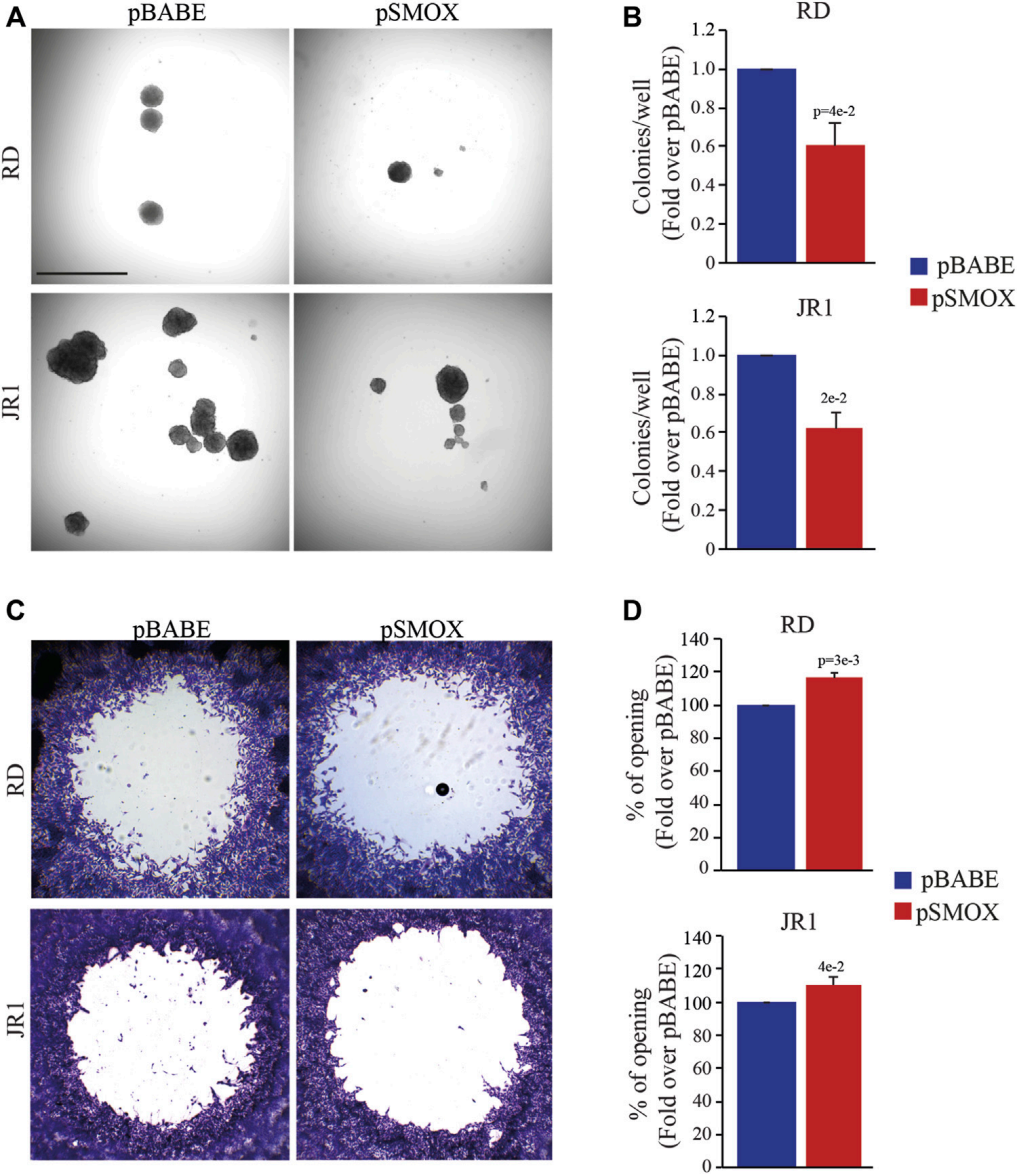
SMOX overexpression affects the in vitro tumorigenic features of FN-RMS cells. **(A)** Representative light microscopy images of soft agar assay of RD and JR1 cells transduced with either pBABE or pSMOX. Scale Bar = 100 μm. **(B)** Histogram of colony number quantification (*n* = 3 biologically independent experiments). Colonies were counted 14 days after seeding. Graphs represent the mean of three independent experiments ±SD, Student two-tailed *t*-Test. Exact *p*-values are reported in the figure. **(C)** Representative phase contrast microscopy images of the migration assays at 24 h after detection zone creation in RD and JR1 cells. **(D)** The histograms depict the measurements of the detection zone of pSMOX, expressed as fold change over control pBABE. Graphs represent the mean of three independent experiments ±SD, Student two-tailed *t*-Test. Exact *p*-values are reported in the figure.

Since the tumorigenicity correlates strictly with the ability of cancer cells to proliferate in the absence of adhesion to extracellular matrix (ECM) proteins, we tested the effect of forced SMOX expression on anchorage-independent growth through soft agar assay considered an *in vitro* surrogate of the *in vivo* tumorigenicity testing. Results indicate that forcing the expression of SMOX reduces the ability of RD and IR1 cells to form colonies with respect to control cells (40 ± 8% and 38 ± 6% reduction, respectively) (Figures 4A, B). In addition, SMOX overexpression decreased the migration of RD and JR1 cells by 16 ± 5% and 11 ± 6%, respectively, as compared with pBABE) (Figures 4C, D).

We also noticed that SMOX upregulation significantly reduces the capability of RD and JR1 cells to grow in 3D spheroids. Specifically, the diameter of spheroids (calculated on Calcein-stained live cells) decreased by 18 ± 2.8% and 20 ± 1.8%, respectively, in SMOX-overexpressing RD and JR1 cells compared to pBABE (Supplementary Figures S3A, B). Moreover, SMOX overexpression induced the appearance of a propidium iodide (PI)-positive dead cell population in spheroids structures from both cell lines, suggesting pro-death effects (Supplementary Figures S3A, B).

Altogether, these findings suggest that SMOX overexpression impairs *in vitro* tumorigenicity of FN-RMS cells by reducing anchorage-independent growth, migration ability and cell growth in 3D.

### SMOX overexpression induces DNA damage in FN-RMS cells

**FIGURE 5:**
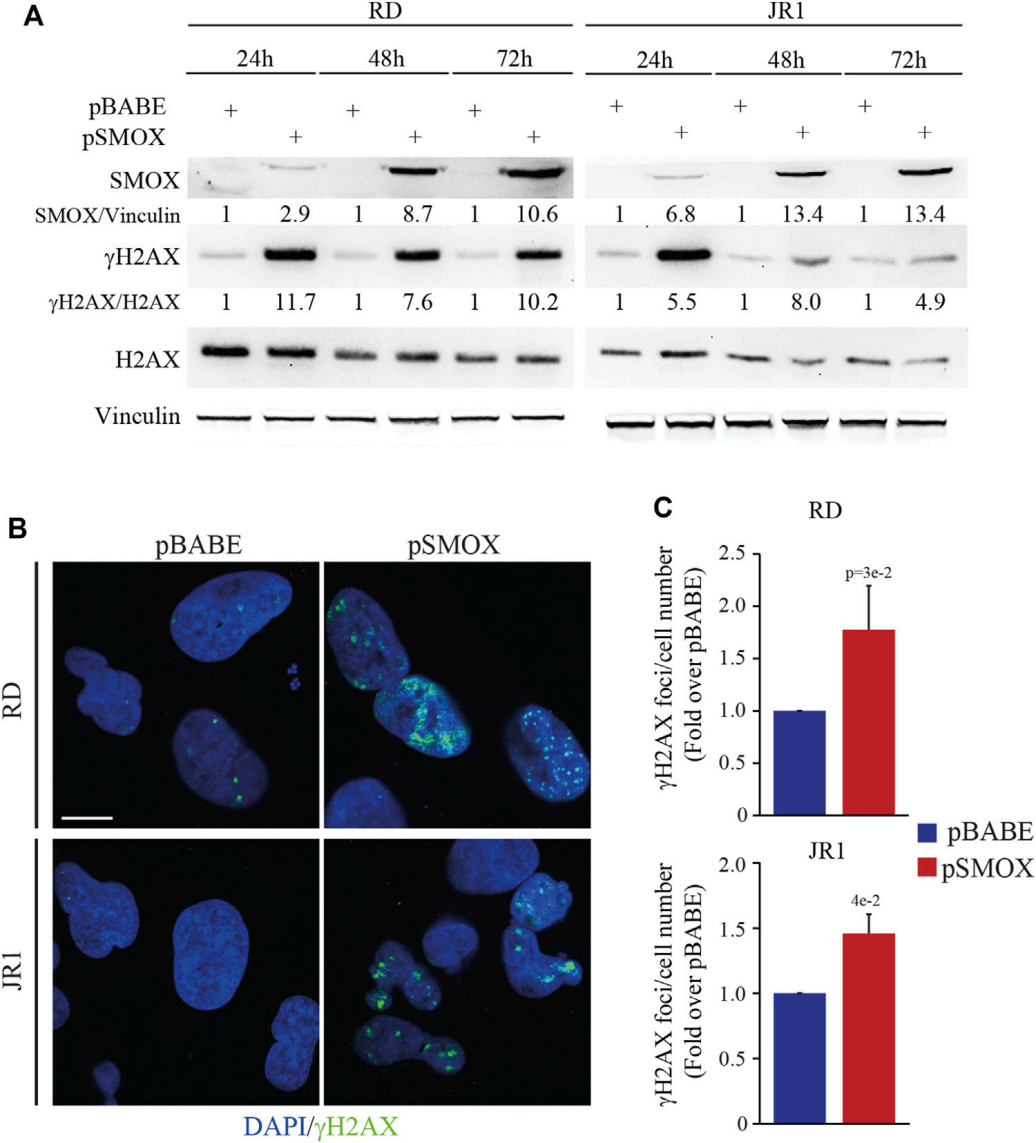
SMOX overexpression in FN-RMS cell lines induces DNA damage. **(A)** Representative western blot (*n* = 3 independent biological replicates) depicting the induction of DNA damage on SMOX overexpressing-RD and -JR1 cells at the reported time points. SMOX, γH2AX and total H2AX protein levels were detected. SMOX and γH2AX levels were normalized to Vinculin and total H2AX protein levels, respectively, and expressed as fold increase over pBABE values. **(B)** Representative immunofluorescence images of γH2AX (green) (*n* = 3 independent biological replicates) in SMOX overexpressing-RD and -JR1 cells 24 h post infection. DAPI (blue) was used as nuclear counterstain. Images were taken using confocal microscopy with a ×60 oil immersion objective lens. Scale Bar = 10 μm. **(C)** Histograms γH2AX foci per cell number in RD and JR1 after 24 h of SMOX overexpression and expressed as fold increase over pBABE values. Graph represents the mean of three independent experiments ±SD, Student two-tailed *t*-Test. Exact *p*-values are reported in the figure.

*In vitro* and *in vivo* studies linked SMOX enzyme activity to DNA damage and cell death (Chaturvedi et al., 2015; Sierra et al., 2020). To gain insights into the anti-proliferative pro-death activity of SMOX, we analyzed the phosphorylation of H2AX histone (γH2AX), a biomarker for double-strand breaks (DSBs), in RD and JR1 cells at 24, 48, and 72 h post-infection with pSMOX or pBABE. Forced expression of SMOX resulted in the upregulation of γH2AX protein levels suggesting an increase in DNA damage (Figure 5A). Consistent with these data, the number of γH2AX foci augmented significantly 24 h post-infection with SMOX high expression (1.8 ± 0.4 and 1.5 ± 0.1 fold increase in RD and JR1, respectively) compared to pBABE (Figures 5B, C). These results suggest that SMOX overexpression behaves as an inducer of DNA damage in FN-RMS cells.

### SMOX overexpression increases irradiation-dependent DNA damage in FN-RMS cells

Based on the results obtained in FN-RMS cells, and given the DNA-damaging properties of radiotherapy, we evaluated whether SMOX overexpression could enhance the sensitivity of FN-RMS cells to irradiation (IR), which is a first-line therapeutic approach in RMS (Petragnano et al., 2020). To this end, 24 h after infection with pSMOX and pBABE, RD and JR1 cells were irradiated with a single dose of 4 Gy and processed 3 h later.

**FIGURE 6:**
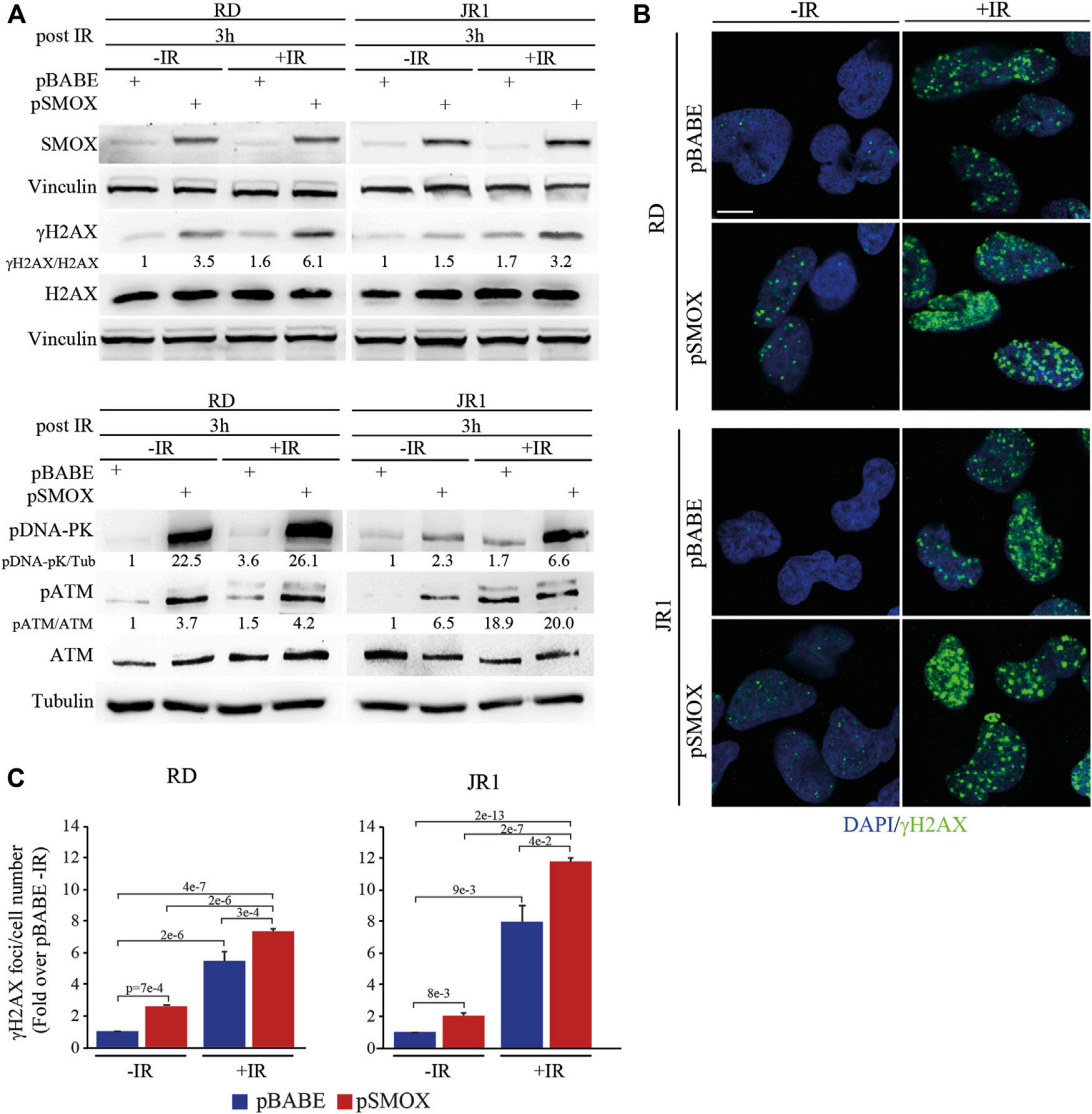
DNA damage induced by SMOX overexpression synergizes with irradiation in FN-RMS cell lines. **(A)** Representative western blot (*n* = 2 independent biological replicates) depicting the effect of irradiation (4Gy), SMOX overexpression and their combination on DNA damage. The cells were processed 3 h post irradiation. SMOX, γH2AX, total H2AX, pATM (Ser 1981), total ATM and pDNA-PK (Thr2609) protein levels were detected. SMOX and pDNA-PK were normalized on Vinculin and Tubulin, respectively. γH2AX and pATM were normalized on H2AX and ATM protein levels, respectively. Protein levels were expressed as fold increase over pBABE—IR values. **(B)** Representative immunofluorescence images of γH2AX (green) (*n* = 2 independent biological replicates) in RD and JR1 cells treated as in **(A)**. DAPI (blue) was used as nuclear counterstain. Images were taken using confocal microscopy with a ×60 oil immersion objective lens. Scale Bar = 10 μm. **(C)** Histograms of γH2AX foci per cell number in RD and JR1 cells treated as in **(A)**. Results were expressed as fold increase over pBABE—IR values. Graph represents the mean of three independent experiments ±SD, one-way ANOVA. Exact *p*-values are reported in the figure.

As expected, IR increased the levels of γH2AX in pBABE-infected RD or JR1 cells (5.4 ± 0.6 and 8 ± 1 fold increase in pBABE + IR vs*.* pBABE) (Figure 6A). Strikingly, when the pSMOX-infected FN-RMS cells were irradiated, the levels of γH2AX were further upregulated (2.8 ± 0.1 and 5.7 ± 0.5 fold increase in pSMOX + IR vs*.* pSMOX) (Figure 6A). Similarly, IR augmented the number of γH2AX foci in pSMOX-infected compared to pBABE-infected RD or JR1 cells (1.4 ± 0.2 and 1.5 ± 0.2 fold increase pSMOX + IR vs*.* pBABE + IR in RD and JR1 cells, respectively) (Figures 6B, C).

In agreement with these findings, the levels of phosphorylated/activated proteins of the DSBs repair pathways, such as ATM and DNA-PK, increased in RD and JR1 cells upon forced SMOX expression or IR, as single treatment (Figure 6A). This effect was robustly reinforced when SMOX-overexpressing RD or JR1 cells were irradiated (Figure 6A).

Altogether, these data suggest that SMOX enzyme activity strengthens IR ability of promoting DNA damage in FN-RMS cells.

### SMOX sensitizes FN-RMS cells to irradiation

**FIGURE 7:**
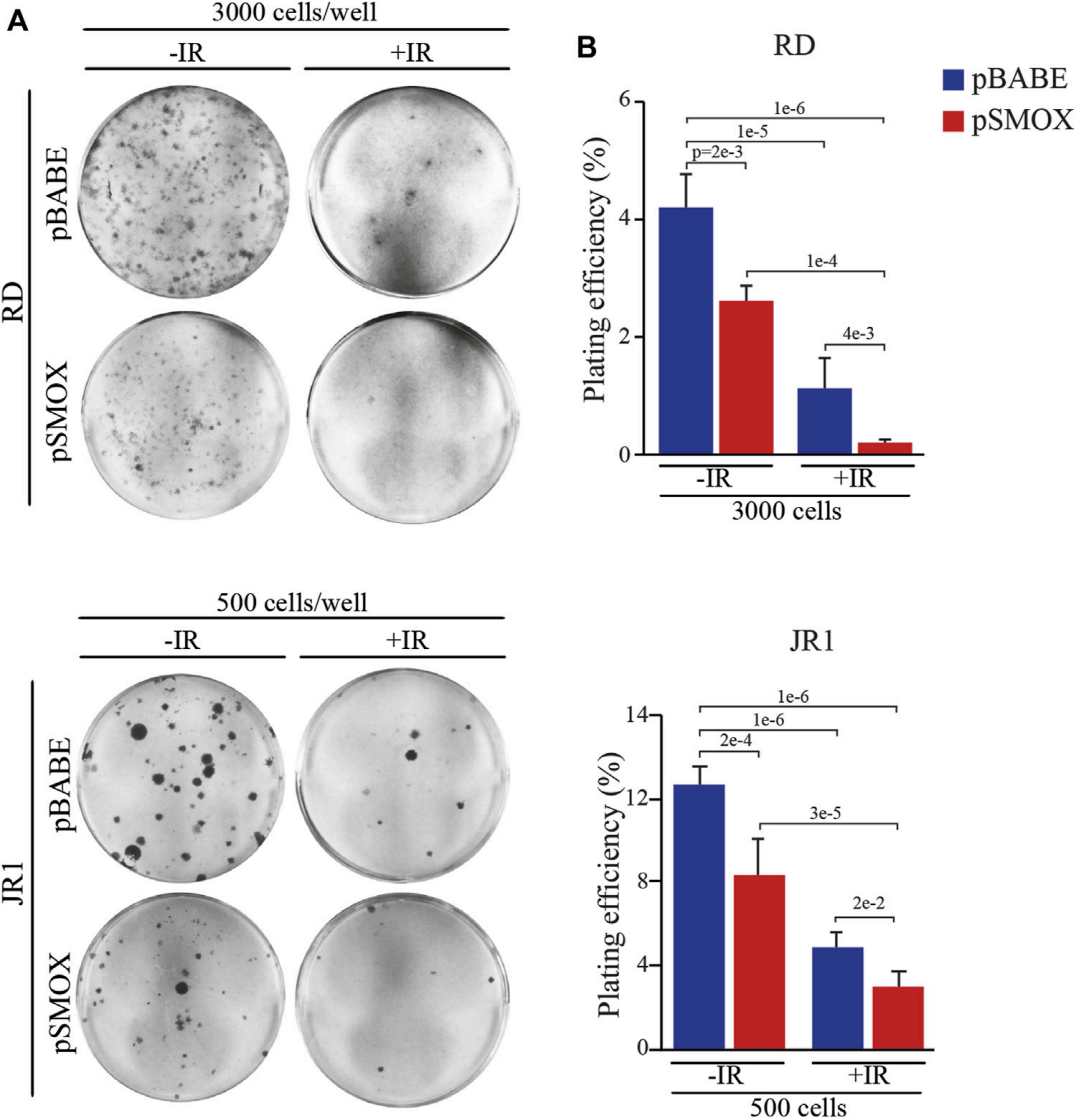
SMOX overexpression radiosensitizes FN-RMS cell lines. **(A)** Representative pictures of RD and JR1 colonies stained with crystal violet 12 days post seeding. FN-RMS cells were infected with either pBABE or pSMOX and 24 h later irradiated with 2 Gy. The cells were processed 3 h post irradiation. **(B)** Histograms depicting the plating efficiency (%) of RD and JR1 infected with either pBABE or pSMOX and irradiated (+IR) or not (- IR) with 2 Gy. Graph represents the mean of three independent experiments ±SD, one-way ANOVA. Exact *p*-values are reported in the figure.

Since the residual tumor cells repopulation that forms recurrences depends on the capacity of cells to self-renewal, the effect of forced SMOX expression alone or in combination with IR exposure was tested by clonogenic cell growth. To this end, colony formation assays with SMOX-overexpressing RD and JR1 cells followed or not by 2 Gy IR exposure was performed. Plating efficiency (PE), obtained by the ratio number of colonies/number of cells seeded, was calculated as reported in (Franken et al., 2006). Figures 7A, B show that both pSMOX and IR as single treatments markedly reduced the capability of tumor cells to form colonies compared to non-irradiated control vector cells of about 38 ± 5.5% and 34 ± 11% (pSMOX vs*.* pBABE) and 71 ± 16% and 61 ± 3% (pBABE + IR vs*.* pBABE) in RD and JR1 cells, respectively.

Noteworthy, when combined with forced expression of SMOX, IR hampered colony formation to an extent which was greater than that promoted by the single SMOX-overexpression or by IR alone (Figures 7A, B).

We, next, examined combined treatments on the growth of cells cultured in 3D. SMOX-overexpressing and control RD and JR1 cells were treated or not with a 4 Gy single dose of IR and the diameters of spheroids were calculated on Calcein-stained live cells. Results indicated that SMOX overexpression significantly reduces the growth compared to pBABE (16 ± 3% and 19 ± 0.5% decrease in RD and JR1 cells, respectively) (Supplementary Figure S4). IR alone also affected 3D growth by reducing the spheres diameter (21 ± 4% and 17 ± 1% decrease in RD and JR1 cells, respectively) (Supplementary Figure S4). Combination of SMOX overexpression and IR further lowered the spheroids diameter in both cell lines compared to each single treatment of 96 ± 0.4% and 24 ± 0.7% (pSMOX + IR vs*.* pBABE + IR), 96 ± 0.02% and 22 ± 0.9% (pSMOX + IR vs*.* pSMOX) and 97 ± 0.1% and 37 ± 0.3% (pSMOX + IR vs*.* pBABE) in RD and JR1 cells, respectively. Notably, the appearance of a PI-positive dead cell population (in red) after single treatments was strongly increased by the combination in both cell lines.

Altogether, these data indicate that SMOX overexpression enhances IR efficacy against FN-RMS cells.

## Discussion

Given the importance of the dysregulation of PAs metabolism in cancer (Novita Sari et al., 2021; Holbert et al., 2022), in an attempt to gain insights into the molecular underpinnings of RMS we have investigated the functions of SMOX, a catabolic enzyme regulating SPM oxidation to SPD.

We show here that patients of the FN-RMS subtype with low levels of SMOX have a worse prognosis compared to those with high expression. Moreover, analysis of a cancer dependency map dataset revealed no dependency on SMOX of FN-RMS cell lines after deletion of the gene. In this regard, we have also observed that 2 cell lines derived from high-risk (recurrent and p53-mutated) FN-RMS tumors display lower protein and mRNA levels of SMOX as compared to normal proliferating myoblasts, used as healthy control.

Then, we have examined the relationship between SMOX expression and myogenesis, which is of particular interest in the RMS context being this tumor most likely originated from normal myogenic-derived cells unable to differentiate. Previous work has demonstrated that SMOX is important in skeletal muscle cells differentiation and in the maintenance of healthy muscle tissue (Cervelli et al., 2009; Ceci et al., 2017) and that a reduction of SMOX levels in differentiated muscles promotes skeletal fiber atrophy (Bongers et al., 2015; Reinoso-Sánchez et al., 2020). However, we have found SMOX expression downregulated during drug-induced differentiation of FN-RMS cells (Yohe et al., 2018). Despite the putative binding sites of MYOD on SMOX promoter and potential enhancer regions previously reported (Tenente et al., 2017), we have found that SMOX expression is indirectly repressed by the MYOD transcription factor in FN-RMS cells. This finding is corroborated by a significant negative correlation between the expression of the SMOX and MYOD genes in FN-RMS patients.

In addition, we have observed that at 24 h after infection of FN-RMS cells with pSMOX, the levels of SPD increase, those of SPM are reduced, while PUT remains unchanged. This result can be explained, at least in part, considering that the catabolic conversion of SPD to PUT is insufficiently activated at this early time point. This is confirmed by the low basal transcript levels of spermidine/spermine-N-acetyltransferase (SAT1) and the high levels of polyamine oxidase (PAOX) which are detectable in our cell lines as compared to HSMM (data not shown). In fact, SPD must be acetylated by SAT1 to be converted to PUT by PAOX (Supplementary Figure S1). Moreover, only the acetylated forms of PAs produced by SAT1 are excreted from the cells, this possibly explaining why the SPD formed by SMOX accumulates at those experimental conditions. On the other hand, we cannot exclude that, being SAT1 highly induced when PAs cell content rises, its expression could be upregulated at late time points resulting in changes in PUT levels (Murray Stewart et al., 2018). However, albeit SAT1 is a p53-direct target, in the p53-mutated/inactivated FN-RMS cell lines used in this work, it cannot be activated by p53 in response to DNA damage (Ou et al., 2016; Tse et al., 2022).

We have also observed that forced expression of SMOX promotes cell cycle arrest and apoptosis of FN-RMS cells, with a concomitant reduction of their tumorigenic features, such as anchorage-independent growth. These results are in agreement with the previous finding that an increase in SPD intracellular levels induces reactive oxygen species (ROS) production (i.e., H_2_O_2_), this leading to the death of neuroblastoma, breast or lung cancer cells (McCloskey et al., 1996; Pledgie et al., 2005; Lodeserto et al., 2022). In this regard, it has to be considered an increase in SPD intracellular levels can cause cancer cell death also in a ROS-independent manner through Bim activation (Guo et al., 2020).

Consistently, forced expression of SMOX significantly reduces the growth of FN-RMS cells cultured in 3D as spheroids in the presence of serum, an experimental condition which mimics tumor cell proliferation *in vivo*. Noteworthy, SMOX overexpression can also hamper FN-RMS cells migration. This is likely to be due to SMOX enzyme capability of reducing SPM levels, as the latter can favor cellular locomotion (Yuan et al., 2000).

In accordance with previous studies indicating that SMOX induction parallels DNA damage in a variety of cancer cell types (Amendola et al., 2005; Bianchi et al., 2007; Chaturvedi et al., 2011; Murray-Stewart et al., 2017), here we have also shown that forced SMOX expression is accompanied by the appearance of DNA damage markers in FN-RMS cells.

Then, based on the fact that IR induces DNA damage and triggers apoptosis in RMS cells as well as other cancer cell types (Codenotti et al., 2021; Rossetti et al., 2021; Liu et al., 2022), that SPM has a protective effect against DNA damage induced by IR (Douki et al., 2000), and that SPM levels drop when SMOX expression is forced, we have evaluated whether combining IR with SMOX overexpression could be a good strategy to kill FN-RMS cells.

We have found that the combination of SMOX overexpression and IR has a dramatic effect on the colony-forming ability of FN-RMS cells compared to each single approach. Under a molecular point of view, SMOX-overexpressing FN-RMS cells, when irradiated, show increased γH2AX protein levels and foci formation, compared to single treatments, which is in line with results in neuroblastoma cells (Bianchi et al., 2007). Generally, with an increase of DNA damage, a raise of the catalytic activity of the two enzymes DNA-PKCs and ATM, respectively upstream of Non-Homologous End-Joining (NHEJ) and Homologous Recombination (HR) DSBs repair pathways, is observed (Toulany, 2019). Accordingly, we have shown that SMOX overexpression further enhances the DNA-PKCs and ATM phosphorylation/activation status induced by IR in FN-RMS cells 3 h post-irradiation, a time interval commonly known to be sufficient for DNA repair in normal but not in cancer cells (Tarnawski et al., 2002; Marcu et al., 2004; Begg et al., 2011). These findings clearly demonstrate that SMOX overexpression sensitizes RMS cells to IR treatment *in vitro* partly by amplifying the response of NHEJ and HR signaling.

As compared to each individual treatment, SMOX overactivation and IR synergize at impairing the survival and growth of FN-RMS cells even when they form *in vitro* 3D structures mimicking what occurs *in vivo*.

Under a translational point of view, PA analogues have been developed such as BESpm, BENSpm, and DENSpm have been developed which augment intracellular levels of both SAT1 and SMOX, hence leading to PAs depletion ultimately resulting in tumor-selective cytotoxicity (Casero et al., 2018). However, results of clinical trials carried out with these drugs are controversial (Hector et al., 2008; Cervelli et al., 2010; Tummala et al., 2011), probably due to the poor absorbability of the first-generation PA analogues by cancer cells. To improve uptake reaching an intracellular elevated concentration, PAs-nanocarriers are being exploited. Among them, Nano11047 effectively induces SAT1 and SMOX enzymatic activities and hampers cancer cell survival and growth in preclinical models (Reddy et al., 1998; Kuo et al., 2009; Smith et al., 2011; Murray-Stewart et al., 2017) and is now being evaluated in clinical trials (www.clinicaltrials.org). It has been used on lung cancer cells showing that it is able to reduce cell growth *in vitro* and PAs biosynthesis and to induce SAT1 and SMOX activities supporting the feasibility of the approach. Nevertheless however, search for efficient and selective SMOX or PAOX inhibitors of is still ongoing (Di Paolo et al., 2019). Recently, nanoSPD have been used to carry SPM into neuroblastoma cells, impairing cell growth and potentiating the effects of nanofenretidine *in vitro* (Lodeserto et al., 2022).

SMOX expression or function has been correlated to tumorigenesis in a context-related manner. Specifically, an increase in SMOX activity parallels the progression of prostate, liver and gastric carcinoma (Goodwin et al., 2008; Chaturvedi et al., 2015; Murray-Stewart et al., 2016; Hu et al., 2018), while high polyamine catabolic enzyme levels have been positively related to a good prognosis in breast cancer patients (Wallace et al., 2000; Cervelli et al., 2010). Herein, we have shown that SMOX has an anti-tumorigenic role in FN-RMS. Although over time SMOX-induced DNA damage could promote pro-tumorigenic local inflammation, as observed in neuroblastoma cells (Amendola et al., 2005; Bianchi et al., 2007; Amendola et al., 2013; Amendola et al., 2014), our results support the use of SMOX induction to radio-sensitize FN-RMS cells that express low levels of the enzyme.

## Data Availability

Publicly available datasets were analyzed in this study. This data can be found here: https://www.ebi.ac.uk/arrayexpress/experiments/E-TABM-1202/; https://depmap.org/portal/; GSE52529; GSE85170.

## References

[B1] Amendola R. Bellini A. Cervelli M. Degan P. Marcocci L. Martini F. (2005). Direct oxidative DNA damage, apoptosis and radio sensitivity by spermine oxidase activities in mouse neuroblastoma cells. Biochim. Biophys. Acta 1755, 15–24. 10.1016/J.BBCAN.2005.02.002 15907589

[B2] Amendola R. Cervelli M. Fratini E. Sallustio D. E. Tempera G. Ueshima T. (2013). Reactive oxygen species spermine metabolites generated from amine oxidases and radiation represent a therapeutic gain in cancer treatments. Int. J. Oncol. 43, 813–820. 10.3892/ijo.2013.2013 23857253

[B3] Amendola R. Cervelli M. Tempera G. Fratini E. Varesio L. Mariottini P. (2014). Spermine metabolism and radiation-derived reactive oxygen species for future therapeutic implications in cancer: An additive or adaptive response. Amino Acids 46, 487–498. 10.1007/s00726-013-1579-9 23999645

[B4] Arruabarrena-Aristorena A. Zabala-Letona A. Carracedo A. (2018). Oil for the cancer engine: The cross-talk between oncogenic signaling and polyamine metabolism. Sci. Adv. 4, eaar2606. 10.1126/sciadv.aar2606 29376126 PMC5783676

[B5] Basu Roy U. K. Rial N. S. Kachel K. L. Gerner E. W. (2008). Activated K-RAS increases polyamine uptake in human colon cancer cells through modulation of caveolar endocytosis. Mol. Carcinog. 47, 538–553. 10.1002/MC.20414 18176934 PMC2515561

[B6] Begg A. C. Stewart F. A. Vens C. (2011). Strategies to improve radiotherapy with targeted drugs. Nat. Rev. Cancer 11, 239–253. 10.1038/nrc3007 21430696

[B7] Bergeron C. Jenney M. De Corti F. Gallego S. Merks H. Glosli H. (2021). Embryonal rhabdomyosarcoma completely resected at diagnosis: The European paediatric Soft tissue sarcoma Study Group RMS2005 experience. Eur. J. Cancer 146, 21–29. 10.1016/J.EJCA.2020.12.025 33567392

[B8] Bianchi M. Bellini A. Cervelli M. Degan P. Marcocci L. Martini F. (2007). Chronic sub-lethal oxidative stress by spermine oxidase overactivity induces continuous DNA repair and hypersensitivity to radiation exposure. Biochim. Biophys. Acta - Mol. Cell Res. 1773, 774–783. 10.1016/j.bbamcr.2007.01.014 17363080

[B9] Bongers K. S. Fox D. K. Kunkel S. D. Stebounova L. V. Murry D. J. Pufall M. A. (2015). Spermine oxidase maintains basal skeletal muscle gene expression and fiber size and is strongly repressed by conditions that cause skeletal muscle atrophy. Am. J. Physiol. Metab. 308, E144–E158. 10.1152/ajpendo.00472.2014 PMC4297781 25406264

[B10] Camero S. Vitali G. Pontecorvi P. Ceccarelli S. Anastasiadou E. Cicchetti F. (2021). DNMT3A and DNMT3B targeting as an effective radiosensitizing strategy in embryonal rhabdomyosarcoma. Cells 10, 2956. 10.3390/CELLS10112956 34831178 PMC8616246

[B11] Casero R. A. Murray Stewart T. Pegg A. E. (2018). Polyamine metabolism and cancer: Treatments, challenges and opportunities. Nat. Rev. Cancer 18, 681–695. 10.1038/s41568-018-0050-3 30181570 PMC6487480

[B12] Cassandri M. Pomella S. Rossetti A. Petragnano F. Milazzo L. Vulcano F. (2021). MS-275 (entinostat) promotes radio-sensitivity in PAX3-FOXO1 rhabdomyosarcoma cells. Int. J. Mol. Sci. 22, 10671. 10.3390/IJMS221910671 34639012 PMC8508838

[B13] Ceci R. Duranti G. Leonetti A. Pietropaoli S. Spinozzi F. Marcocci L. (2017). Adaptive responses of heart and skeletal muscle to spermine oxidase overexpression: Evaluation of a new transgenic mouse model. Free Radic. Biol. Med. 103, 216–225. 10.1016/J.FREERADBIOMED.2016.12.040 28043891

[B14] Cervelli M. Angelucci E. Germani F. Amendola R. Mariottini P. (2014b). Inflammation, carcinogenesis and neurodegeneration studies in transgenic animal models for polyamine research. Amino Acids 46, 521–530. 10.1007/s00726-013-1572-3 23933909

[B15] Cervelli M. Bellavia G. Fratini E. Amendola R. Polticelli F. Barba M. (2010). Spermine oxidase (SMO) activity in breast tumor tissues and biochemical analysis of the anticancer spermine analogues BENSpm and CPENSpm. BMC Cancer 10, 555. 10.1186/1471-2407-10-555 20946629 PMC3027604

[B16] Cervelli M. Fratini E. Amendola R. Bianchi M. Signori E. Ferraro E. (2009). Increased spermine oxidase (SMO) activity as a novel differentiation marker of myogenic C2C12 cells. Int. J. Biochem. Cell Biol. 41, 934–944. 10.1016/j.biocel.2008.09.009 18852063

[B17] Cervelli M. Pietropaoli S. Signore F. Amendola R. Mariottini P. (2014a). Polyamines metabolism and breast cancer: State of the art and perspectives. Breast Cancer Res. Treat. 148, 233–248. 10.1007/S10549-014-3156-7 25292420

[B18] Cervelli M. Salvi D. Polticelli F. Amendola R. Mariottini P. (2013). Structure–function relationships in the evolutionary framework of spermine oxidase. J. Mol. Evol. 76, 365–370. 10.1007/s00239-013-9570-3 23828398

[B19] Chaturvedi R. Asim M. Romero–Gallo J. Barry D. P. Hoge S. de Sablet T. (2011). Spermine oxidase mediates the gastric cancer risk associated with *Helicobacter pylori* CagA. Gastroenterology 141, 1696–708.e1–2. 10.1053/j.gastro.2011.07.045 21839041 PMC3202654

[B20] Chaturvedi R. De Sablet T. Asim M. Piazuelo M. B. Barry D. P. Verriere T. G. (2015). Increased Helicobacter pylori-associated gastric cancer risk in the Andean region of Colombia is mediated by spermine oxidase. Oncogene 34, 3429–3440. 10.1038/ONC.2014.273 25174398 PMC4345146

[B21] Chen Y. Zhuang H. Chen X. Zheqi S. H. I. Wang X. (2018). Spermidine-induced growth inhibition and apoptosis via autophagic activation in cervical cancer. Oncol. Rep. 39, 2845–2854. 10.3892/OR.2018.6377 29693131

[B22] Codenotti S. Marampon F. Triggiani L. Bonù M. L. Magrini S. M. Ceccaroli P. (2021). Caveolin-1 promotes radioresistance in rhabdomyosarcoma through increased oxidative stress protection and DNA repair. Cancer Lett. 505, 1–12. 10.1016/j.canlet.2021.02.005 33610729

[B23] D’Amico D. Antonucci L. Di Magno L. Coni S. Sdruscia G. Macone A. (2015). Non-canonical hedgehog/AMPK-mediated control of polyamine metabolism supports neuronal and medulloblastoma cell growth. Dev. Cell 35, 21–35. 10.1016/J.DEVCEL.2015.09.008 26460945 PMC4607931

[B24] Davicioni E. Finckenstein F. G. Shahbazian V. Buckley J. D. Triche T. J. Anderson M. J. (2006). Identification of a PAX-FKHR gene expression signature that defines molecular classes and determines the prognosis of alveolar rhabdomyosarcomas. Cancer Res. 66, 6936–6946. 10.1158/0008-5472.CAN-05-4578 16849537

[B25] Di Paolo M. L. Cervelli M. Mariottini P. Leonetti A. Polticelli F. Rosini M. (2019). Exploring the activity of polyamine analogues on polyamine and spermine oxidase: Methoctramine, a potent and selective inhibitor of polyamine oxidase. J. Enzyme Inhib. Med. Chem. 34, 740–752. 10.1080/14756366.2019.1584620 30829081 PMC6407594

[B26] Douki T. Bretonniere Y. Cadet J. (2000). Protection against radiation-induced degradation of DNA bases by polyamines. Radiat. Res. 153, 29–35. 10.1667/0033-7587(2000)153[0029:parido]2.0.co;2 10630975

[B27] Franken N. A. P. Rodermond H. M. Stap J. Haveman J. van Bree C. (2006). Clonogenic assay of cells *in vitro* . Nat. Protoc. 1, 2315–2319. 10.1038/nprot.2006.339 17406473

[B28] Goodwin A. C. Jadallah S. Toubaji A. Lecksell K. Hicks J. L. Kowalski J. (2008). Increased spermine oxidase expression in human prostate cancer and prostatic intraepithelial neoplasia tissues. Prostate 68, 766–772. 10.1002/pros.20735 18302221 PMC3065872

[B29] Gryder B. E. Yohe M. E. Chou H. C. Zhang X. Marques J. Wachtel M. (2017). PAX3-FOXO1 establishes myogenic super enhancers and confers BET bromodomain vulnerability. Cancer Discov. 7, 884–899. 10.1158/2159-8290.CD-16-1297 28446439 PMC7802885

[B30] Guo Y. Ye Q. Deng P. Cao Y. He D. Zhou Z. (2020). Spermine synthase and MYC cooperate to maintain colorectal cancer cell survival by repressing Bim expression. Nat. Commun. 11, 3243. 10.1038/S41467-020-17067-X 32591507 PMC7320137

[B31] Hector S. Tummala R. Kisiel N. D. Diegelman P. Vujcic S. Clark K. (2008). Polyamine catabolism in colorectal cancer cells following treatment with oxaliplatin, 5-fluorouracil and N1, N11 diethylnorspermine. Cancer Chemother. Pharmacol. 62, 517–527. 10.1007/s00280-007-0633-2 17987291

[B32] Holbert C. E. Cullen M. T. Casero R. A. Stewart T. M. (2022). Polyamines in cancer: Integrating organismal metabolism and antitumour immunity. Nat. Rev. Cancer 22, 467–480. 10.1038/s41568-022-00473-2 35477776 PMC9339478

[B33] Hu T. Sun D. Zhang J. Xue R. Janssen H. L. A. Tang W. (2018). Spermine oxidase is upregulated and promotes tumor growth in hepatocellular carcinoma. Hepatol. Res. 48, 967–977. 10.1111/hepr.13206 29923661

[B34] Igarashi K. Kashiwagi K. (2019). The functional role of polyamines in eukaryotic cells. Int. J. Biochem. Cell Biol. 107, 104–115. 10.1016/J.BIOCEL.2018.12.012 30578954

[B35] Kahana C. (2016). Protein degradation, the main hub in the regulation of cellular polyamines. Biochem. J. 473, 4551–4558. 10.1042/BCJ20160519C 27941031

[B36] Kucharzewska P. Welch J. E. Svensson K. J. Belting M. (2009). The polyamines regulate endothelial cell survival during hypoxic stress through PI3K/AKT and MCL-1. Biochem. Biophys. Res. Commun. 380, 413–418. 10.1016/J.BBRC.2009.01.097 19250631

[B37] Kuo W.-L. Das D. Ziyad S. Bhattacharya S. Gibb W. J. Heiser L. M. (2009). A systems analysis of the chemosensitivity of breast cancer cells to the polyamine analogue PG-11047. BMC Med. 7, 77. 10.1186/1741-7015-7-77 20003408 PMC2803786

[B38] Li J. Meng Y. Wu X. Sun Y. (2020). Polyamines and related signaling pathways in cancer. Cancer Cell Int. 20, 539. 10.1186/S12935-020-01545-9 33292222 PMC7643453

[B39] Liu R. Bian Y. Liu L. Liu L. Liu X. Ma S. (2022). Molecular pathways associated with oxidative stress and their potential applications in radiotherapy (Review). Int. J. Mol. Med. 49, 65. 10.3892/ijmm.2022.5121 35293589 PMC8989428

[B40] Lodeserto P. Rossi M. Blasi P. Farruggia G. Orienti I. (2022). Nanospermidine in combination with nanofenretinide induces cell death in neuroblastoma cell lines. Pharmaceutics 14, 1215. 10.3390/PHARMACEUTICS14061215 35745787 PMC9229898

[B41] Manni A. Grove R. Kunselman S. Aldaz M. (1995). Involvement of the polyamine pathway in breast cancer progression. Cancer Lett. 92, 49–57. 10.1016/0304-3835(95)03763-M 7757960

[B42] Marcu L. van Doorn T. Olver I. (2004). Modelling of post-irradiation accelerated repopulation in squamous cell carcinomas. Phys. Med. Biol. 49, 3767–3779. 10.1088/0031-9155/49/16/021 15446804

[B43] McCloskey D. E. Yang J. Woster P. M. Davidson N. E. Casero R. A. (1996). Polyamine analogue induction of programmed cell death in human lung tumor cells. Clin. Cancer Res. 2, 441–446. Available at: http://www.ncbi.nlm.nih.gov/pubmed/9816189 . 9816189

[B44] Menna M. Fiorentino F. Marrocco B. Lucidi A. Tomassi S. Cilli D. (2022). Novel non-covalent LSD1 inhibitors endowed with anticancer effects in leukemia and solid tumor cellular models. Eur. J. Med. Chem. 237, 114410. 10.1016/J.EJMECH.2022.114410 35525212

[B45] Murray Stewart T. Dunston T. T. Woster P. M. Casero R. A. (2018). Polyamine catabolism and oxidative damage. J. Biol. Chem. 293, 18736–18745. 10.1074/jbc.TM118.003337 30333229 PMC6290137

[B46] Murray-Stewart T. Ferrari E. Xie Y. Yu F. Marton L. J. Oupicky D. (2017). Biochemical evaluation of the anticancer potential of the polyamine-based nanocarrier Nano11047. PLoS One 12, e0175917. 10.1371/journal.pone.0175917 28423064 PMC5396973

[B47] Murray-Stewart T. Sierra J. C. Piazuelo M. B. Mera R. M. Chaturvedi R. Bravo L. E. (2016). Epigenetic silencing of miR-124 prevents spermine oxidase regulation: Implications for Helicobacter pylori-induced gastric cancer. Oncogene 35, 5480–5488. 10.1038/onc.2016.91 27041578 PMC5050049

[B48] Novita Sari I. Setiawan T. Seock Kim K. Toni Wijaya Y. Won Cho K. Young Kwon H. (2021). Metabolism and function of polyamines in cancer progression. Cancer Lett. 519, 91–104. 10.1016/j.canlet.2021.06.020 34186159

[B49] Ou Y. Wang S.-J. Li D. Chu B. Gu W. (2016). Activation of SAT1 engages polyamine metabolism with p53-mediated ferroptotic responses. Proc. Natl. Acad. Sci. 113, E6806–E6812. 10.1073/pnas.1607152113 27698118 PMC5098629

[B50] Pegg A. E. (2016). Functions of polyamines in mammals. J. Biol. Chem. 291, 14904–14912. 10.1074/JBC.R116.731661 27268251 PMC4946908

[B51] Perrone C. Pomella S. Cassandri M. Pezzella M. Milano G. M. Colletti M. (2022). MET inhibition sensitizes rhabdomyosarcoma cells to NOTCH signaling suppression. Front. Oncol. 12, 835642. 10.3389/FONC.2022.835642 35574376 PMC9092259

[B52] Petragnano F. Pietrantoni I. Di Nisio V. Fasciani I. Del Fattore A. Capalbo C. (2020). Modulating the dose-rate differently affects the responsiveness of human epithelial prostate- and mesenchymal rhabdomyosarcoma-cancer cell line to radiation. Int. J. Radiat. Biol. 96, 823–835. 10.1080/09553002.2020.1739774 32149569

[B53] Pledgie A. Huang Y. Hacker A. Zhang Z. Woster P. M. Davidson N. E. (2005). Spermine oxidase SMO(PAOh1), Not N1-acetylpolyamine oxidase PAO, is the primary source of cytotoxic H2O2 in polyamine analogue-treated human breast cancer cell lines. J. Biol. Chem. 280, 39843–39851. 10.1074/JBC.M508177200 16207710

[B54] Pomella S. Sreenivas P. Gryder B. E. Wang L. Milewski D. Cassandri M. (2021). Interaction between SNAI2 and MYOD enhances oncogenesis and suppresses differentiation in Fusion Negative Rhabdomyosarcoma. Nat. Commun. 12, 192. 10.1038/S41467-020-20386-8 33420019 PMC7794422

[B55] Reddy V. K. Valasinas A. Sarkar A. Basu H. S. Marton L. J. Frydman B. (1998). Conformationally restricted analogues of 1 N, 12 N -bisethylspermine: Synthesis and growth inhibitory effects on human tumor cell lines. J. Med. Chem. 41, 4723–4732. 10.1021/jm980172v 9822543

[B56] Reinoso-Sánchez J. F. Baroli G. Duranti G. Scaricamazza S. Sabatini S. Valle C. (2020). Emerging role for linear and circular spermine oxidase RNAs in skeletal muscle physiopathology. Int. J. Mol. Sci. 21, 8227. 10.3390/ijms21218227 33153123 PMC7663755

[B57] Rossetti A. Petragnano F. Milazzo L. Vulcano F. Macioce G. Codenotti S. (2021). Romidepsin (FK228) fails in counteracting the transformed phenotype of rhabdomyosarcoma cells but efficiently radiosensitizes, *in vitro* and *in vivo*, the alveolar phenotype subtype. Int. J. Radiat. Biol. 97, 943–957. 10.1080/09553002.2021.1928786 33979259

[B58] Shern J. F. Chen L. Chmielecki J. Wei J. S. Patidar R. Rosenberg M. (2014). Comprehensive genomic analysis of rhabdomyosarcoma reveals a landscape of alterations affecting a common genetic axis in fusion-positive and fusion-negative tumors. Cancer Discov. 4, 216–231. 10.1158/2159-8290.CD-13-0639 24436047 PMC4462130

[B59] Shern J. F. Selfe J. Izquierdo E. Patidar R. Chou H.-C. Song Y. K. (2021). Genomic classification and clinical outcome in rhabdomyosarcoma: A report from an international consortium. J. Clin. Oncol. 39, 2859–2871. 10.1200/JCO.20.03060 34166060 PMC8425837

[B60] Sierra J. C. Piazuelo M. B. Luis P. B. Barry D. P. Allaman M. M. Asim M. (2020). Spermine oxidase mediates Helicobacter pylori-induced gastric inflammation, DNA damage, and carcinogenic signaling. Oncogene 39, 4465–4474. 10.1038/S41388-020-1304-6 32350444 PMC7260102

[B61] Silvestrini R. Daidone M. G. Valagussa P. Di Fronzo G. Mezzanotte G. Bonadonna G. (1989). Cell kinetics as a prognostic indicator in node-negative breast cancer. Eur. J. Cancer Clin. Oncol. 25, 1165–1171. 10.1016/0277-5379(89)90410-0 2767105

[B62] Skapek S. X. Ferrari A. Gupta A. A. Lupo P. J. Butler E. Shipley J. (2019). Rhabdomyosarcoma. Nat. Rev. Dis. Prim. 5, 1. 10.1038/s41572-018-0051-2 30617281 PMC7456566

[B63] Smith M. A. Maris J. M. Lock R. Kolb E. A. Gorlick R. Keir S. T. (2011). Initial testing (stage 1) of the polyamine analog PG11047 by the pediatric preclinical testing program. Pediatr. Blood Cancer 57, 268–274. 10.1002/pbc.22797 21360650 PMC3115432

[B64] Snezhkina A. V. Krasnov G. S. Lipatova A. V. Sadritdinova A. F. Kardymon O. L. Fedorova M. S. (2016). The dysregulation of polyamine metabolism in colorectal cancer is associated with overexpression of c-myc and C/EBPβ rather than enterotoxigenic Bacteroides fragilis infection. Oxid. Med. Cell. Longev. 2016, 2353560. 10.1155/2016/2353560 27433286 PMC4940579

[B65] Tarnawski R. Fowler J. Skladowski K. Świerniak A. Suwiński R. Maciejewski B. (2002). How fast is repopulation of tumor cells during the treatment gap? Int. J. Radiat. Oncol. 54, 229–236. 10.1016/S0360-3016(02)02936-X 12182996

[B66] Tenente I. M. Hayes M. N. Ignatius M. S. McCarthy K. Yohe M. Sindiri S. (2017). Myogenic regulatory transcription factors regulate growth in rhabdomyosarcoma. Elife 6, e19214. 10.7554/ELIFE.19214 28080960 PMC5231408

[B67] Toulany M. (2019). Targeting DNA double-strand break repair pathways to improve radiotherapy response. Genes (Basel). 10, 25. 10.3390/genes10010025 30621219 PMC6356315

[B68] Tse R. T.-H. Ding X. Wong C. Y.-P. Cheng C. K.-L. Chiu P. K.-F. Ng C.-F. (2022). The association between spermidine/spermine N1-acetyltransferase (SSAT) and human malignancies. Int. J. Mol. Sci. 23, 5926. 10.3390/ijms23115926 35682610 PMC9179984

[B69] Tummala R. Diegelman P. Hector S. Kramer D. L. Clark K. Zagst P. (2011). Combination effects of platinum drugs and N1, N11 diethylnorspermine on spermidine/spermine N1-acetyltransferase, polyamines and growth inhibition in A2780 human ovarian carcinoma cells and their oxaliplatin and cisplatin-resistant variants. Cancer Chemother. Pharmacol. 67, 401–414. 10.1007/s00280-010-1334-9 20443003 PMC3028085

[B70] Wallace H. M. Duthie J. Evans D. M. Lamond S. Nicoll K. M. Heys S. D. (2000). Alterations in polyamine catabolic enzymes in human breast cancer tissue. Clin. Cancer Res. 6, 3657–3661. Available at: http://www.ncbi.nlm.nih.gov/pubmed/10999758 . 10999758

[B71] Wallace H. M. Fraser A. V. Hughes A. (2003). A perspective of polyamine metabolism. Biochem. J. 376, 1–14. 10.1042/BJ20031327 13678416 PMC1223767

[B72] Wang C. Ruan P. Zhao Y. Li X. Wang J. Wu X. (2017). Spermidine/spermine N1-acetyltransferase regulates cell growth and metastasis via AKT/β-catenin signaling pathways in hepatocellular and colorectal carcinoma cells. Oncotarget 8, 1092–1109. 10.18632/ONCOTARGET.13582 27901475 PMC5352037

[B73] Wang L. Hensch N. R. Bondra K. Sreenivas P. Zhao X. R. Chen J. (2021). SNAI2-Mediated repression of BIM protects rhabdomyosarcoma from ionizing radiation. Cancer Res. 81, 5451–5463. 10.1158/0008-5472.CAN-20-4191 34462275 PMC8669772

[B74] Yohe M. E. Gryder B. E. Shern J. F. Song Y. K. Chou H. C. Sindiri S. (2018). MEK inhibition induces MYOG and remodels super-enhancers in RAS-driven rhabdomyosarcoma. Sci. Transl. Med. 10, eaan4470. 10.1126/SCITRANSLMED.AAN4470 29973406 PMC8054766

[B75] Yuan Q. Viar M. J. Ray R. M. Johnson L. R. (2000). Putrescine does not support the migration and growth of IEC-6 cells. Am. J. Physiol. Liver Physiol. 278, G49–G56. 10.1152/ajpgi.2000.278.1.G49 10644561

