# Supplementary material for: Spermine oxidase induces DNA damage and sensitizes fusion negative rhabdomyosarcoma cells to irradiation
Source: Front Cell Dev Biol. 2023 Jan 23;11:1061570. doi: 10.3389/fcell.2023.1061570 (PMC9900442; doi:10.3389/fcell.2023.1061570)
Supplement: Supplementary file 1 [file DataSheet1.pdf]

Supplementary Material

1. Supplementary Figures

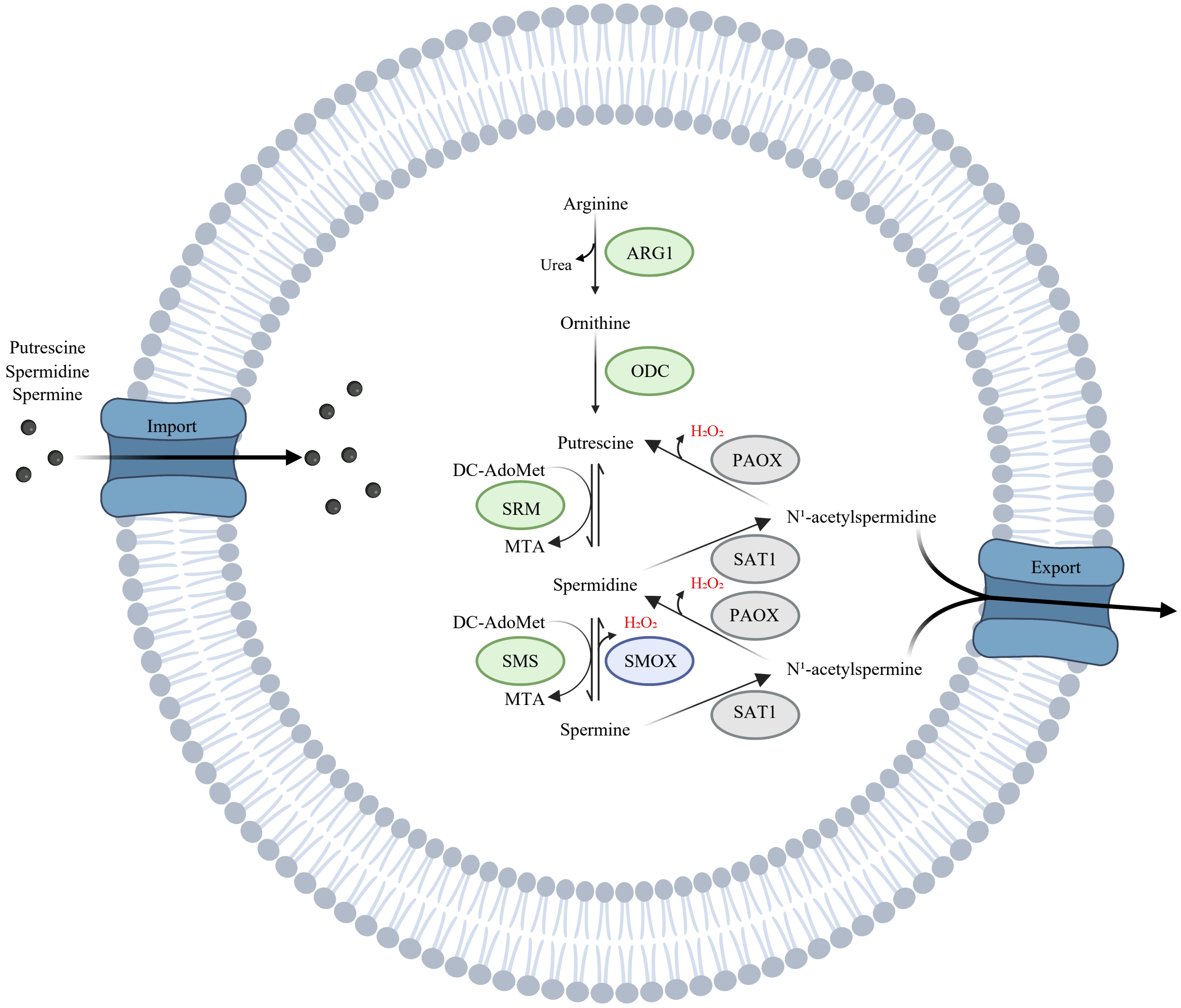

Supplementary Figure 1. Mammalian PAs metabolism. Schematic representation of PAs metabolism showing enzyme network and substrate interconversion pathways (figure realized with BioRender, https://biorender.com/). ARG1: Arginase 1; ODC: Ornithine Decarboxylase; SRM; Spermidine Synthase; SMS: Spermine Synthase; SMOX: Spermine Oxidase; SAT1: Spermidine/Spermine N1-Acetyltransferase 1; PAOX: Polyamine Oxidase.

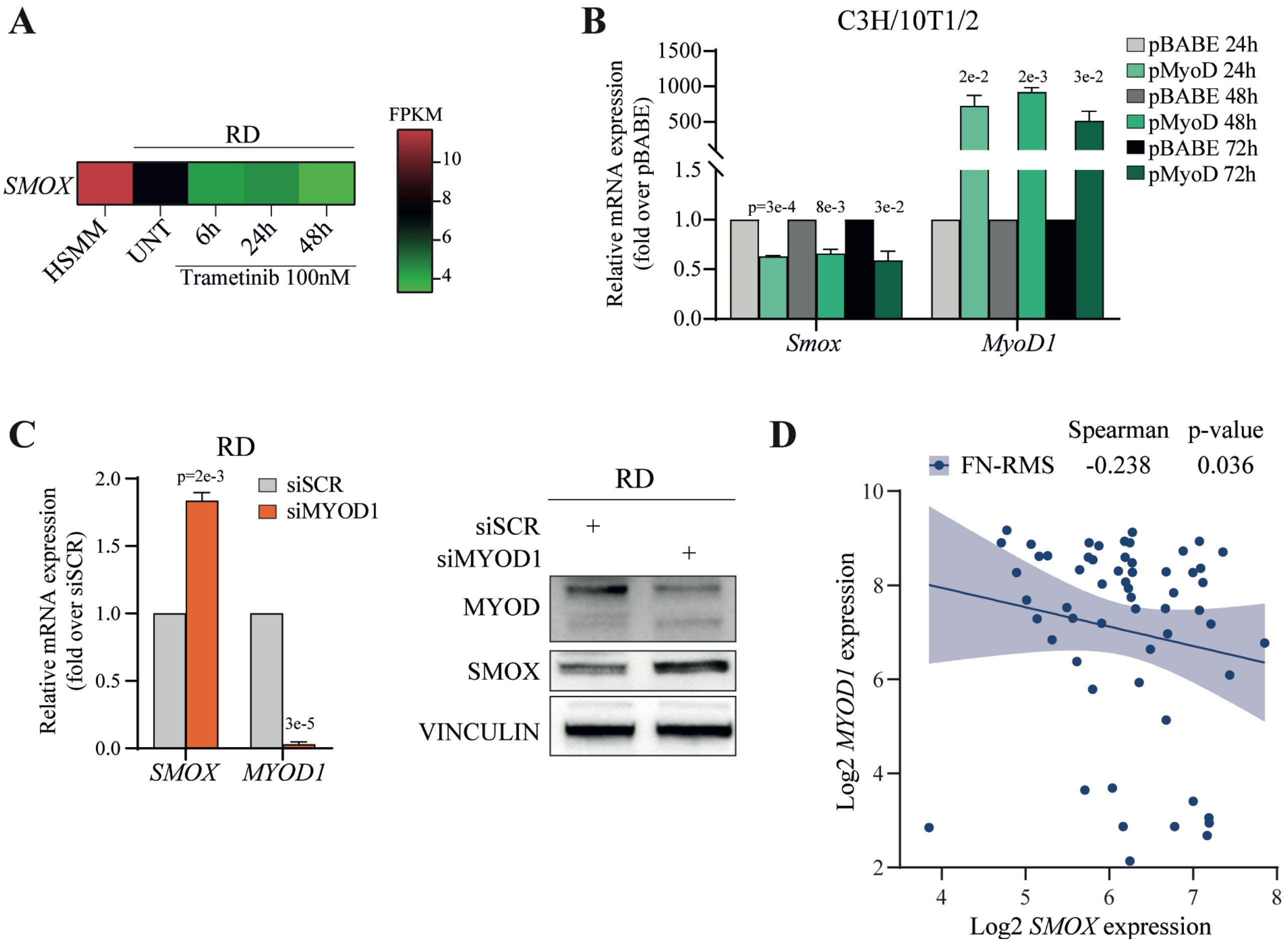

Supplementary Figure 2. ***SMOX*** **expression decreases in a Trametinib differentiation model and is negatively regulated by MYOD. (A)** Heatmap depicting RNA-seq data of *SMOX* levels in Human Skeletal Muscle Myoblasts (HSMM) and in RD treated with 100 nM of Trametinib at the reported time points. UNT: untreated; FPKM: fragments per kilobase million reads. **(B)** *Smox* and *MyoD1* mRNA levels (qRT-PCR) of C3H/10T1/2 murine fibroblast overexpressing either MyoD or control vector (pBABE) at the reported time points. Gene levels were expressed as fold increase over pBABE values. Graph represents the mean of three independent experiments ± SD, Student two-tailed T-Test. Exact p-values are reported in the figure. **(C)** SMOX and MYOD1 mRNA levels (qRT-PCR, left panel) and protein levels (right panel) of RD cells transfected for 48h with either a control siRNA (siSCR) or a validated MYOD1 targeting siRNA (siMYOD1). Gene levels were expressed as fold increase over siSCR values. Graph represents the mean of three independent experiments ± SD, Student two-tailed T-Test. Exact p-values are reported in the figure. **(D)** Spearman correlation analysis of *MYOD1* and *SMOX* expression in FN-RMS (n=58) patients. Linear regression Spearman coefficient and p-value are reported in the figure.

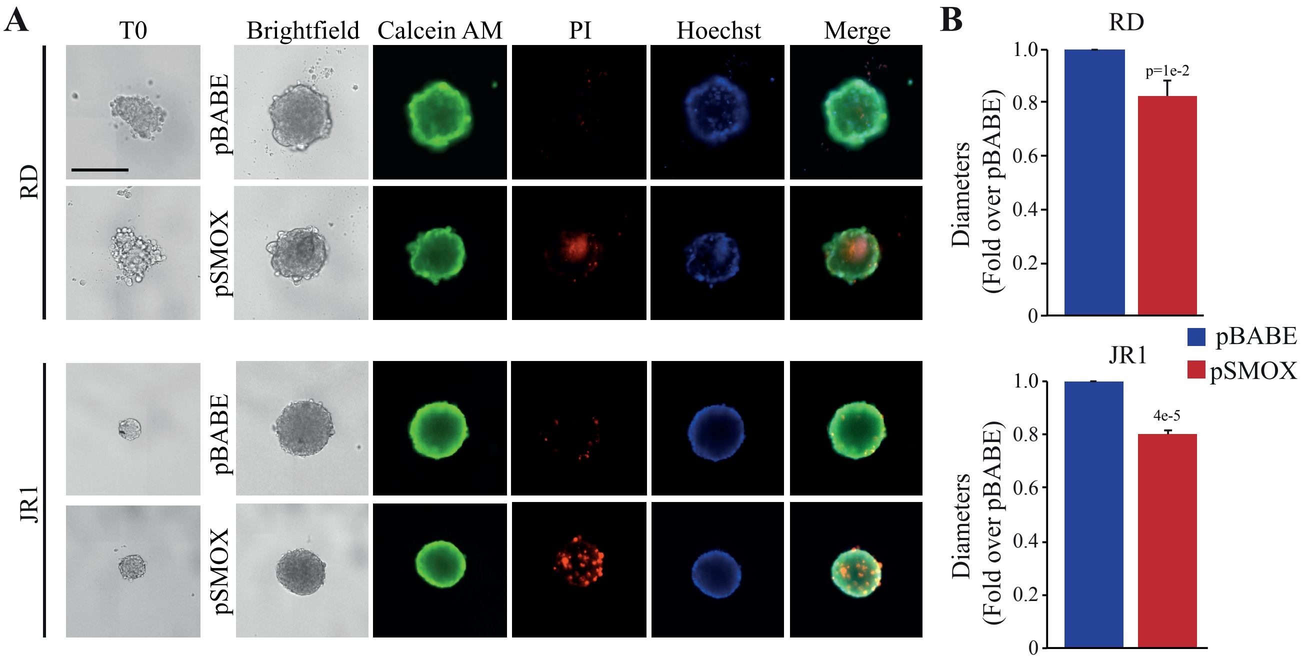

Supplementary Figure 3. SMOX overexpression hampers 3D cell growth in FN-RMS cells. **(A)** Representative images of SMOX overexpressing-RD and -JR1 tumor spheroids 6 days post seeding. Spheroid diameters were calculated, and 3D tumor spheroids were stained with Calcein AM (green), Propidium Iodide (red) and Hoechst (blue). Scale bars = 500 µm. **(B)** Histogram of spheroid diameter quantification in RD and JR1 infected with either pBABE or pSMOX. Graphs represent the mean of three independent experiments ± SD, Student two-tailed T-Test. Exact p-values are reported in the figure.

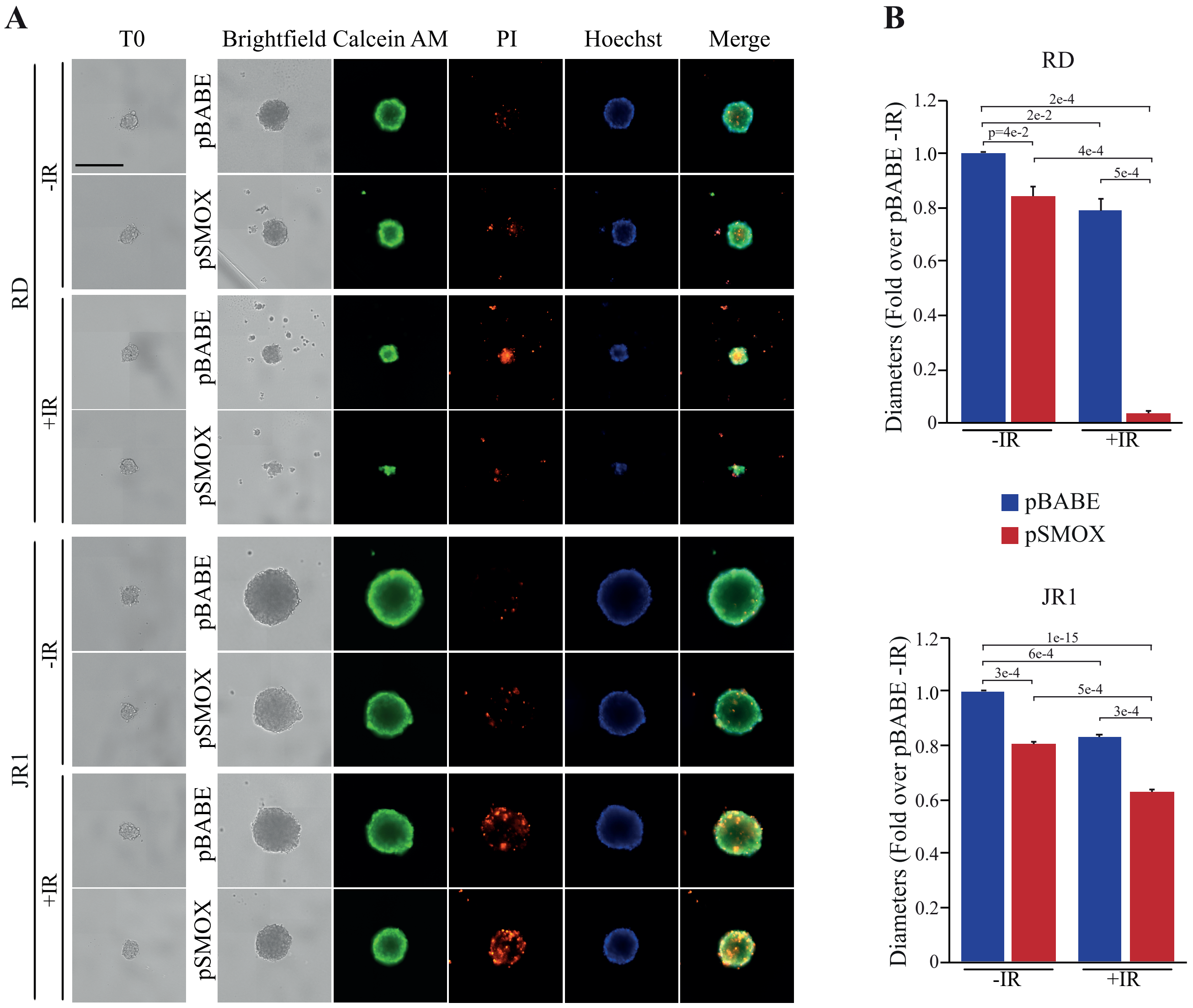

Supplementary figure 4. SMOX overexpression synergizes with ionizing radiation hampering 3D cell growth in FN-RMS cells. (A) Representative images of pBABE and pSMOX overexpressing-RD and -JR1 tumor spheroids 6 days post seeding. pBABE and pSMOX cells were seeded 24h post infection and irradiated with 4 Gy. After additional 3h the cells were seeded. After 6 days spheroid diameters were calculated and 3D tumor spheroids were stained with Calcein AM (green), Propidium Iodide (red) and Hoechst (blue). Scale bars = 500 µm. **(B)** Histograms of spheroid diameter quantification of pBABE and pSMOX overexpressing-RD and -JR1, irradiated (+ IR) or not (- IR) with 4 Gy. Graph represents the mean of three independent experiments ± SD, one-way ANOVA. Exact p-values are reported in the figure.
